# Outcomes of Digital Training for Community Health Workers in Low- and Middle-Income Countries: Scoping Review

**DOI:** 10.2196/82772

**Published:** 2026-05-19

**Authors:** Tapiwa A Tembo, Nora Ellen Rosenberg, Firaol Ayele, Saeed Ahmed, Linda-Gail Bekker

**Affiliations:** 1 Baylor College of Medicine Children's Foundation Malawi, Off Mzimba Road, P/Bag B-397, Lilongwe, Malawi, 265 999362440; 2 Department of Health Behavior, Gillings School of Global Public Health, University of North Carolina at Chapel Hill, Chapel Hill, NC, United States; 3 Department of Pediatrics, Baylor College of Medicine, Houston, TX, United States; 4 The Desmond Tutu HIV Centre, Institute of Infectious Disease and Molecular Medicine, Faculty of Health Sciences, University of Cape Town, Cape Town, South Africa

**Keywords:** community health worker, digital training: blended learning, eLearning, LMIC, lay health worker

## Abstract

**Background:**

Community health workers (CHWs) play an important role in delivering essential health services in low- and middle-income countries (LMICs). Training CHWs using digital approaches is on the rise. Although scoping and systematic reviews of digital training have been conducted for medical professionals in high-income countries (HICs), none have been conducted with lay professionals in LMICs, a population with different considerations.

**Objective:**

This review describes the characteristics of digital training for CHWs and identifies their impact on health services outcomes in LMICs.

**Methods:**

A scoping review approach based on Arksey and O’Malley’s guiding principles was used to retrieve, review, and analyze existing literature. We searched 10 foremost databases using keywords and Medical Subject Headings terms for CHWs, LMICs, and digital learning to identify primary, peer-reviewed studies published up to and including November 26, 2024. An updated search of studies in all the databases was conducted on January 12, 2026, by the research team. No registries were searched. Articles that focused on the provision of digital or blended learning training for CHWs working in LMICs in any disease domain evaluating a learning, implementation, or clinical outcome met the eligibility criteria. Two reviewers (TAT and FA) screened the articles at the title and abstract levels and at full-text review. Study details, study designs, training attributes, technology and CHW descriptions, and outcomes were abstracted using a data-charting form. Descriptive analysis was conducted of the population, training characteristics, and reported outcomes. The PRISMA (Preferred Reporting Items for Systematic Reviews and Meta-Analyses) guidelines for reporting scoping reviews were used.

**Results:**

A total of 892 articles were retrieved and screened for eligibility, of which 18 original articles met the inclusion criteria. Most (n=13) were conducted in Asia. Most (n=15) used nonrandomized study designs. The most common attributes included synchronous (n=8), accessible in the community (n=14), use of smartphones (n=6), and accessible online (n=9). The majority reported learning outcomes (n=14), about half reported implementation outcomes (n=10), and only one reported clinical outcomes (n=1). The learning outcomes focused on knowledge gained and were mostly positive. The implementation outcomes included CHW’s acceptability and feasibility to use the digital training approach. The clinical outcome was effectiveness.

**Conclusions:**

We found few evaluations of digital training for CHWs in LMICs, in spite of a proliferation of such trainings. Digital trainings had a broad range of attributes. Many evaluations had knowledge, acceptability, and feasibility outcomes. However, other learning outcomes (eg, attitudes and skills), implementation outcomes (eg, appropriateness and fidelity), and clinical outcomes were rare. Most lacked experimental designs. Although the existing evidence suggests that digital training can impact knowledge in lay health workers in LMICs, more rigorous studies with a broader range of outcomes are needed.

## Introduction

Low- and middle-income countries (LMICs) face a shortage of professional health care workers (HCWs) [1]. To overcome this gap, lay HCWs or community health workers (CHWs) have been recruited and trained to carry out an increasing array of tasks [2]. CHWs are laypeople working within their own community, performing functions related to health care delivery and health promotion, but have not received formal professional or paraprofessional certificates or degrees [3]. CHWs are often trained for specific tasks such as HIV testing, disease screening, or provision of immunization and have been recognized as critical role-players within the primary health care setting [4], where task shifting to CHWs has been shown to be a cost-effective method of service delivery scale-up [5,6]. When provided with the correct resources, training, and support, CHWs have improved accessibility to basic health services, resulting in better health outcomes [7,8].

The World Health Organization (WHO) recommends that CHWs receive regular training and supervision to fulfill their roles successfully [9]. To increase the competency of CHWs in health care provision, there is a need for more effective, higher-quality, and easily accessible health training [10]. The design of these training approaches would ideally also minimize the burden on an already strained health care system by limiting the time CHWs spend away from workstations while attending training required to improve service delivery.

The use of digital technology in CHW education may help overcome these challenges. In line with this, the increasing availability of technology could address the shortcomings of in-service training provision to CHWs. Digital training may provide more accessible, standardized, relevant, timely, and affordable solutions [11,12]. Moreover, digital training may provide flexibility that would allow participants to balance their tasks and learning endeavors effectively [13,14]. Given the widespread availability of technology devices, digital training by itself or in combination with face-to-face classroom training (ie, blended learning) has been widely used for medical education in a variety of settings [5,9,15-17]. These training approaches have facilitated improvements in knowledge, skills, overall competence, and clinical performance of health professionals across various health care settings, such as clinicians, nurses, and public health practitioners [18,19]. In addition, the COVID-19 pandemic necessitated and reinforced the practice of digital training for health workers, including lay cadres [14,20]. However, regardless of the benefits associated with digital training, the evidence surrounding these trainings for CHWs has not yet been synthesized.

Given that there are over 8 million CHWs in LMICs [21,22] globally, ensuring that they all receive optimal and appropriate training efficiently and cost-effectively is a challenge but also a critical priority. Digital solutions may present a viable option given that digital training can be effective in high-income countries (HICs) with professional cadres. However, evidence on the impact of digital training on capacity-building for CHWs on factors including knowledge, skills enhancement, and improved health care needs to be explored and understood. It is important to know whether these solutions can be applied to nonprofessional cadres and LMIC settings where there are limited digital skills and infrastructures [23,24], and whether they will be found to be acceptable, feasible, and effective. To develop and implement successful digital training programs for CHWs in resource-constrained settings, a greater understanding is needed of the training attributes and the extent to which these training modalities enhance CHW capacity. The Kirkpatrick evaluation framework articulates four levels of training outcomes: (1) participant reaction to the training program experience; (2) learning outcomes; (3) participant’s change in behavior; and (4) impact on the clinical setting [25]. We undertook this scoping review with this framework in mind. The overall purpose of this scoping review was to synthesize the evidence from the literature surrounding digital and blended learning among lay HCWs in LMICs to highlight areas for future research and implementation. Specifically, we sought to (1) characterize populations using these digital tools by geography and cadres, (2) describe the nature and attributes of blended and digital learning tools, (3) organize types of training outcomes examined by the levels defined in the Kirkpatrick framework, and (4) examine preliminary learning, implementation, and clinical outcomes.

## Methods

### Overview

We conducted a scoping review in accordance with the PRISMA-ScR (Preferred Reporting Items for Systematic Reviews and Meta-Analyses extension for Scoping Reviews) guidelines [26] on the provision of digital and blended learning training for CHWs in LMICs. Our scoping review followed systematic and transparent research steps guided by the framework described by Arksey and O’Malley [27] and updated by Levac et al [28], to characterize the attributes and nature of digital and blended learning training for CHWs in LMICs, as well as the outcomes considered. An internal protocol was developed for the review to define the inclusion and exclusion criteria and the review methods before data extraction.

### Search Strategy

The Cochrane Library and PROSPERO (International Prospective Register of Systematic Reviews) were searched to identify available or ongoing scoping and systematic reviews pertaining to the provision of digital or blended training for CHWs in LMICs. No previous or ongoing relevant reviews were identified.

We then designed a comprehensive search strategy to identify all relevant studies according to the PRISMA-S (PRISMA-Search Extension) guideline [29]. We did not use published search filters. There were no search strategies that were adapted from other studies. A publication date limit was not set. The initial search was conducted on November 26, 2024. Twenty relevant search terms for “Community Health Workers” and 21 search terms for “digital learning” and “blended learning” were developed. These were combined with the World Bank Group 2022 list of LMICs using Boolean operators to develop a master search query. Where appropriate, each index-linked Medical Subject Headings term was expanded to contain all relevant subheadings. In addition, synonyms were searched for each key term, along with wildcards and truncation for free-text words. The following databases were searched to identify primary, peer-reviewed studies published up to and including January 12, 2026, including Cumulated Index in Nursing and Allied Health Literature (CINAHL), Cochrane, Embase, Education Resources Information Center (ERIC), Global Health, Google Scholar, PsycINFO, PubMed, Scopus, and Social Science Research Network (SSRN). The search terms were adjusted accordingly for each database. An updated search of studies, rerunning the searches, was conducted on January 12, 2026, by the research team. Cited references were examined for any relevant studies by browsing reference lists. Study registries were not searched. A full record of the conducted search for each database is provided in the online supplementary material (Table S1 in Multimedia Appendix 1).

### Eligibility Criteria

Studies were included in the review if the population comprised primary participants who were CHWs [3], the concept involved CHWs being trained using digital or blended learning modalities, and the context included the following: (1) the training occurred in a country defined as an LMIC according to the World Bank Group 2022 classification of economies [30]; (2) the primary data were collected; (3) primary focus of the training addressed a communicable or noncommunicable disease domain; and (4) the article reported a learning, implementation, or clinical outcome. Digital learning was defined as a practice of learning using technologies to deliver educational content and training programs [31,32]. Blended learning was an education approach that combines face-to-face and digital learning approaches [33]. The training outcomes needed either to have been evaluated within the same group through pre-post study design or with a control group through randomized or nonrandomized controlled trials.

The scoping review did not restrict based on study design and included both qualitative and quantitative outcomes. Studies had to qualify as an original, full-text research study to be considered for inclusion in the review. Review articles, commentaries, letters, policy briefs, protocols, needs assessments, and conference abstracts were excluded.

### Outcomes

We assessed learning, implementation, or clinical outcomes and noted the studies that reported each outcome. The learning outcomes included knowledge, attitudes, and behaviors. “Knowledge” referred to information acquired through the training. Attitudes focused on confidence to perform tasks. Behaviors focused on whether trainees used what they learned. The implementation outcomes included acceptability, appropriateness, feasibility, and fidelity. Acceptability was defined as the CHW perception that the training approach was agreeable or satisfactory. Appropriateness was the CHW’s perceived fit of the training. Feasibility was defined as the extent to which the training was successfully carried out in the setting. Fidelity was defined as the degree to which the training was delivered as intended. Clinical outcomes included how well the training improved patients’ uptake in clinical practice [34].

### Selection of Sources of Evidence

All articles identified via database searching and other methods, including citation searching were exported into EndNote and imported into Covidence review software, and duplicate references were removed. The screening process was performed according to PRISMA-ScR. Titles and abstracts of all the articles identified in the search were screened by 2 authors (TAT and FA) to determine whether they would be considered relevant for a full-text review. For title and abstract screening, interreviewer reliability with Cohen κ was 0.84. Based on Landis and Koch’s [35] threshold values reference, a κ of ≥0.81 is considered “almost perfect agreement.” The full text of all the articles identified as potentially relevant was then retrieved and reviewed in full against the inclusion and exclusion criteria by both reviewers. All articles that did not meet the eligibility criteria were excluded from the review database, and reasons for exclusion were recorded. At all stages of the review, discrepancies between the authors were resolved via discussion. Where appropriate, the authors of individual papers were contacted for further information. References and other sources were reviewed to identify any other articles for full-text review. A critical appraisal of individual sources of evidence was not performed.

### Data Charting, Extraction, and Synthesis

Data were independently extracted and tabulated by 2 authors (TAT and FA) using a data charting form in a Microsoft Excel spreadsheet that was approved by the research team. The data charting form was used to extract information regarding the population characteristics, training attributes, and identified reported outcomes. The use of a data charting form or table was recommended by Arksey and O’Malley [27] and Levac et al [28]. The data extraction form was piloted by 2 authors on 5 studies prior to use to ensure that all necessary data were captured appropriately. Where necessary, the corresponding authors for relevant studies were contacted via email to clarify aspects of their work prior to final extraction.

The bibliographic data extracted included the first author, title, year of publication, and country in which the study took place. CHW descriptions included CHW cadre name, number of CHWs trained, sex, age, education, disease domain, employment type (full-time or part-time), duty station (community or facility-based), remuneration (presence or absence), and employer. Training features included type of training, modality, synchronicity, venue, device type, availability online or offline, technology medium, duration, number of sessions, and pedagogical approaches. Outcomes included measurements, results, and limitations. The study design information was extracted and categorized into cross-sectional cohort, longitudinal cohort, quasi-experimental, and randomized controlled trials. The analytic approaches were classified into qualitative, quantitative, and mixed methods.

Once the data had been transferred into the spreadsheet, one author (TAT) reviewed the information and organized it into one of the following categories: (1) learning outcomes, (2) implementation outcomes, and (3) clinical outcomes. The descriptions of the included studies were analyzed and organized in tabular format, accompanied by a narrative summary.

## Results

### Search Results

**Figure 1.**
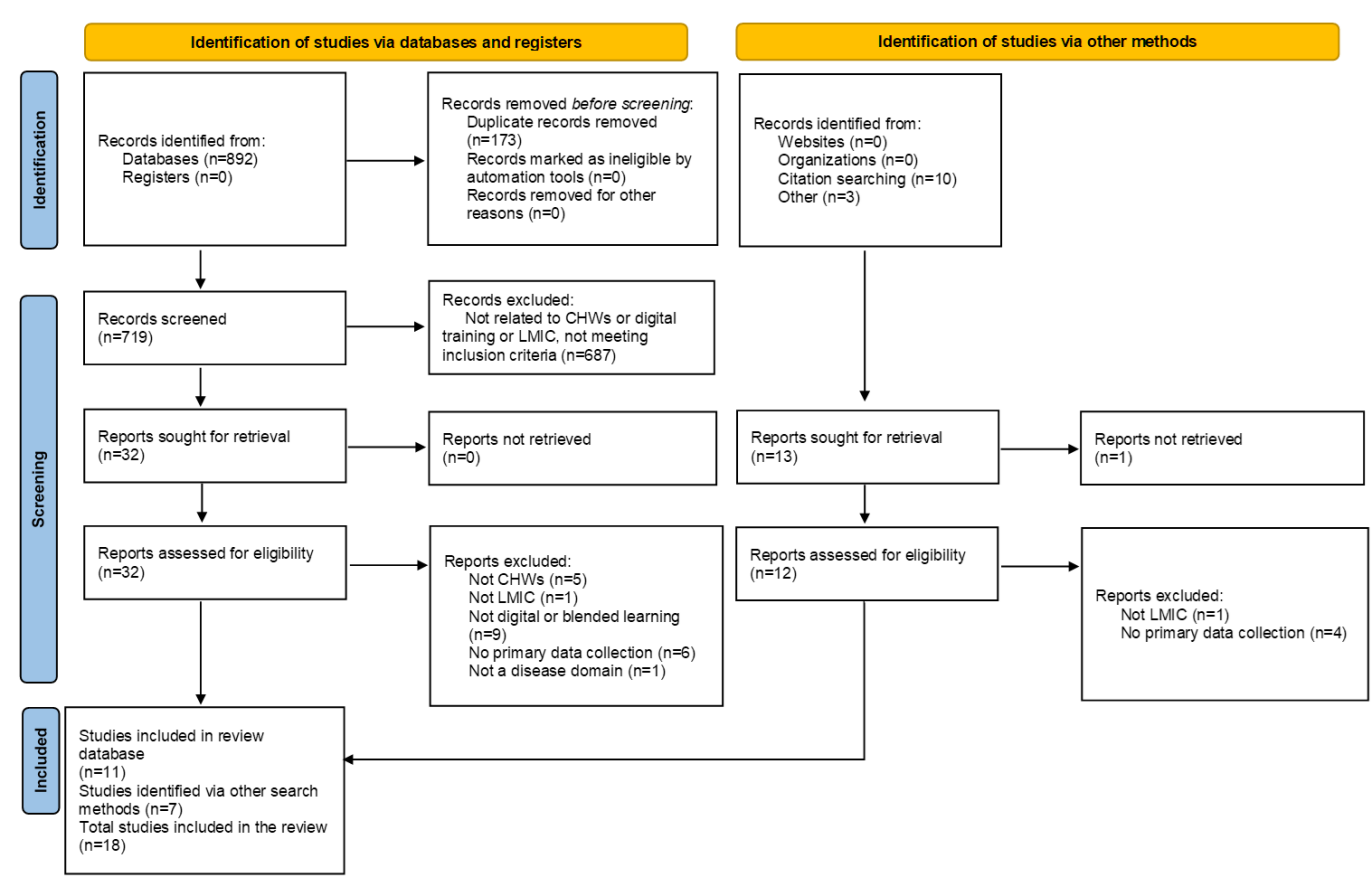
PRISMA (Preferred Reporting Items for Systematic Reviews and Meta-Analyses) flow diagram. CHW: community health worker; LMIC: low- and middle-income country.

The initial search of the 10 databases yielded 892 articles (see online Table S2 in Multimedia Appendix 1). After the exclusion of 173 duplicates, 719 papers were identified for initial screening. After title and abstract screening, 32 studies were identified for full-text review. An additional 13 articles were selected from reference lists and other sources, totaling 45 articles for full-text review. After the full text review, 18 studies [36-53] were identified for data extraction, and 27 were excluded because they did not meet inclusion criteria. Reasons for exclusion at full-text screening can be found in the PRISMA flowchart (Figure 1).

### Characteristics of Included Studies, CHWs, and Training

**Figure 2.**
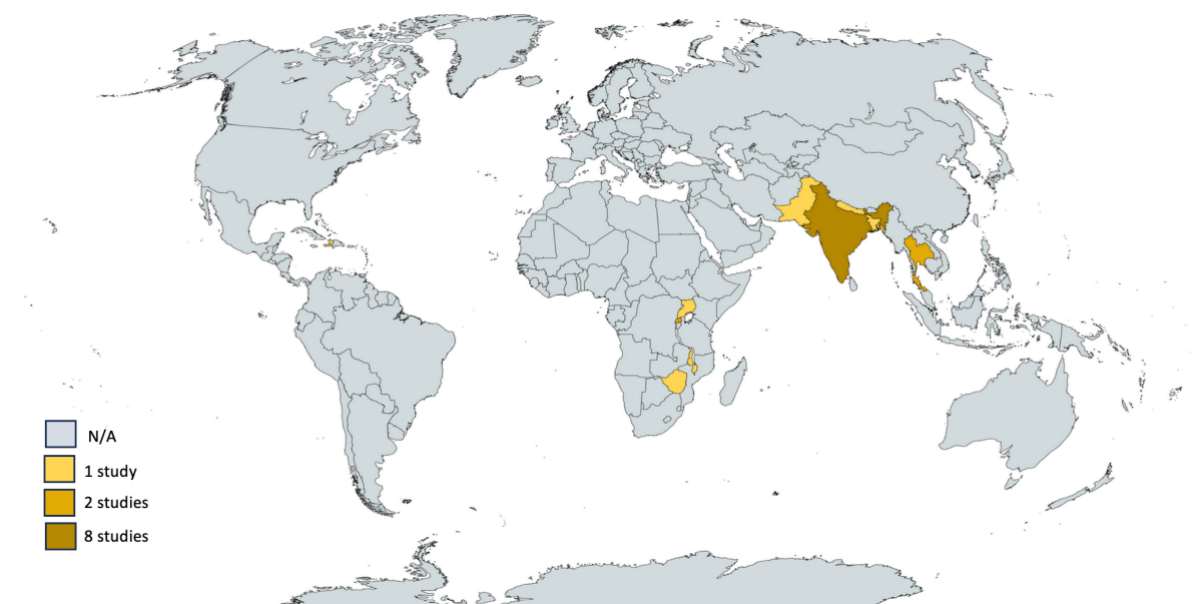
Map of countries where studies included in the scoping review were conducted.

All 18 [36-53] studies in this review were published from 2009 to 2024. A majority of studies were conducted in Asia (n=13) [37-43,45,47,48,51-53], especially in India (n=8) [37,39,41-43,51-53], a few were conducted in Africa (n=5) [36,44,46,49,50], and one study was conducted in the Caribbean [37]. None were conducted in Latin America, Eastern or Southern Europe, or Oceania (Figure 2). The countries where the studies were undertaken are summarized in Table 1.

**Table 1.** Details of studies included in the review.

First author, year	Title	Country	Community health workers name	Number of community health workers	Community health worker sex	Community health worker age	Community health worker education	Disease or domain of focus	Full-time or part-time	Community- or facility-based	Paid or incentivized or not paid	Government or nongovernmental organization
Bertman et al (2019) [36]	Health worker text messaging for blended learning, peer support, and mentoring in pediatric and adolescent HIV/AIDS care: a case study in Zimbabwe	Zimbabwe	Primary counselors (PCs)	293	Male and female	≥25 years	Secondary school education	HIV	Full-time	Both	Paid	Government
Khan et al (2019) [37]	An electronic-based curriculum to train acute care providers in rural Haiti and India	Haiti and India	Acute care providers	Haiti: n=6; India: n=55	Haiti: male and female; India: female	Haiti: mean age 24 years; India: mean age 39 years	Haiti: high school diploma; India: primary education	General acute conditions	Full-time	Community	Not reported	Not reported
Kharel et al (2022) [38]	Training program for female community volunteers to combat COVID 19 in rural Nepal	Nepal	Female community health volunteers (FCHV)	183	Female	Not reported	Not reported	COVID-19	Part-time	Community	Paid	Government
Lakshminarayanan et al (2020) [39]	Delivery of perinatal mental health services by training lay counselors using digital platforms	India	Lay counselors	23	Female	Not reported	Not reported	Perinatal mental health	Part-time	Community	Not reported	Not reported
Limaye et al (2019) [40]	Enhancing the knowledge and behaviors of fieldworkers to promote family planning and maternal, newborn, and child health in Bangladesh through a digital health training package: results from a pilot study	Bangladesh	Community health workers (CHWs; Field workers)	Pre: 306 and post: 265	Female	Mean age pre: 35 (SD 12) years and mean age post: 34 (SD 12) years	Not reported	Maternal, newborn, and child health and family planning	Full-time	Community	Not reported	Government
Muke et al (2019) [41]	Acceptability and feasibility of digital technology for training community health workers to deliver brief psychological treatment for depression in rural India	India	Accredited social health activist (ASHA)	32	Female	24‐45 years	Minimum education level of grade 8	Depression	Part-time	Community	Paid	Government
Muke et al (2020) [42]	Digital training for non-specialist health workers to deliver a brief psychological treatment for depression in primary care in India: findings from a randomized pilot study	India	ASHA	45	Female	≥18 years	Minimum education level of 8th standard	Mental health	Part-time	Community	Paid	Government
Nedungadi et al (2019) [43]	Rural health in digital India: interactive simulations for community health workers	India	CHWs	23	Female	≥18 years	Minimum education level of grade 8	Communicable and noncommunicable diseases and nutrition deficiency	Part-time	Community	Paid	Government
O’Donovan et al (2018) [44]	The use of low-cost Android tablets to train community health workers in Mukono, Uganda, in the recognition, treatment and prevention of pneumonia in children under five: a pilot randomized controlled trial	Uganda	CHWs	163	Male and female	Mean age control group 44.6 (SD 12.5) years; intervention group 43.7 (SD 10.3) years	9 years of education	Pneumonia	Part-time	Community	Not paid	Government
Rahman et al (2019) [45]	Using technology to scale-up training and supervision of community health workers in the psychosocial management of perinatal depression: a non-inferiority, randomized controlled trial	Pakistan	CHWs	80	Female	Mean age control group 35 (SD 8) years; intervention group 36 (SD 7) years	Not reported	Perinatal depression	Full-time	Community	Paid	Government
Sangwa et al (2024) [46]	Using eLearning to improve and retain the knowledge of community health workers in maternal and neonatal health in Rwanda: a cohort study	Rwanda	CHWs	36	Female	≥25 years	Completed primary education	Maternal and newborn health	Part-time	Community	Paid	Government
Sranacharoenpong et al (2009) [47]	Process and outcome evaluation of a diabetes prevention education program for community health care workers in Thailand	Thailand	Community health care workers (CHCW)	69	Male and female	25‐54 years	Diploma level to bachelor's	Diabetes	Full-time	Facility-based	Paid	Government
Sranacharoenpong and Hanning (2012) [48]	Diabetes prevention education program for community health care workers in Thailand	Thailand	Community health care workers (CHCW)	69	Male and female	25‐54 years	Diploma level to bachelor's	Diabetes	Full-time	Facility-based	Paid	Government
Tembo et al (2021) [49]	Pilot-testing a blended learning package for health care workers to improve index testing services in Southern Malawi: an implementation science study	Malawi	CHWs and HIV diagnostic assistants	12	Male and female	20‐42 years	Completed secondary education	HIV	Full-time	Facility-based	Paid	Nongovernmental organization
Willems et al (2021) [50]	Co-creation and evaluation of nationwide remote training service for mental health education of community health workers in Rwanda	Rwanda	CHWs	51,858	Male and Female	20‐50 years	Not reported	Mental health	Part-time	Community	Paid	Government
Yadav et al (2017) [51]	Sangoshthi: empowering community health workers through peer learning in rural India	India	ASHA	40	Female	26‐50 years	Minimum education level of grade 10	Maternal and child health	Part-time	Community	Paid	Government
Yadav D (2017) [52]	Low-cost mobile learning solutions for community health workers	India	ASHA	40	Female	Not reported	Not reported	Maternal and child health	Part-time	Community	Paid	Government
Yadav et al (2019) [53]	LEAP: scaffolding collaborative learning of community health workers in India	India	ASHA	120	Female	25‐45 years	Minimum education level of grade 8	Maternal and child health	Part-time	Community	Paid	Government

Six different CHW terms were identified across the 18 studies, with variations being noted between studies in terms of CHW sex, type of employment contract, duty station, remuneration, and employer. The terms “community health worker” (n=9) [40,43-50] and “accredited social health activist” (n=5) [41,42,51-53] were commonly used. “Lay counselor,” “primary counselor,” “female community health volunteers,” and “acute care provider” were used in one study each. The majority of studies reported employing female CHWs only (n=11) [37-43,45,51-53] and the others employed males and females. The age of CHWs varied from 18 to 54 years. Most CHWs worked part-time (n=10) [38,39,41-44,46,50-53]; were community-based (n=14) [37-46,50-53]; were paid a monthly salary, incentive, or honorarium (n=14) [36,38,41-43,45-53]; and were employed by the government (n=15; Table 1) [36,38,40-44,46-48,50-53].

A total of 4 study designs were identified. Studies were cross-sectional (n=9) [36,37,39-41,43,47,48,50], cohort (n=1) [46], quasi-experimental (n=5) [38,49,51-53], and randomized controlled trials (RCTs, n=3) [42,44,45]. With regard to the analytic approaches applied, most studies were quantitative (n=10) [37-40,44-49] and mixed methods (n=6) [42,43,50-53]. Two used qualitative methods only [36,41].

The training modalities described in the studies were digital learning (n=14) [37-46,50-53] and blended learning (n=4) [36,47-49]. Some training approaches made use of tablets or smartphones (n=6) [36,37,42,44-46]. This was followed by computers (n=5) [40,43,47-49] and basic or feature phones (n=1) [50]. There were some studies that reported the use of multiple technologies (n=5) [39,41,51-53]. The training approaches were all implemented by researchers (n=18) and supported by government (n=2) [46,50] and nongovernmental organizations (n=1) [49]. The training formats were synchronous (n=8) [38,41-43,49,51-53], asynchronous (n=5) [37,40,44,46,50], and both (synchronous and asynchronous) (n=5) [36,39,45,47,48].

The training venue, duration, focus, and pedagogical approaches varied between studies. The majority of the training occurred in the community (n=14) [37-46,50-53] compared to a classroom (n=3) [47-49] or both (n=1) [36]. The trainings focused on the following disease areas: COVID-19 prevention, testing, and management (n=1) [38]; diabetes prevention (n=2) [47,48]; general conditions care or management (n=2) [37,43]; HIV testing services (n=2) [36,49]; maternal and child health (n=5) [40,46,51-53], mental health or depression screening and counseling (n=5) [39,41,42,45,50]; and pneumonia recognition, treatment, and prevention (n=1) [44]. Most of the training was accessible online (n=8) [36,38,39,41-43,46,53] rather than offline (n=6) [37,40,44,45,49,50] or both (n=4) [47,48,51,52]. The duration ranged from a minimum of one day to a maximum of 8 months. They lasted from one to 48 hours. The main pedagogical approaches employed were individual learning (n=5) [39-41,44,50] and group discussion (n=4) [47,48,51,52]. Other approaches included practicing scenarios, modeling, and receiving feedback to reinforce information [36-38,42,43,45,49] (Table 2). Full details of the training are summarized in Table 3

**Table 2.** Digital and blended learning training characteristics.

Study	Type of training	Training modality	Synchronicity (or both)	Health facility or conference or community	Device	Technology medium	Method of training (online vs offline)	Training accessible offline (yes or no)	Time spent in training	Training duration	Number of training sessions or modules	Pedagogical approaches
Bertman et al [36]	In-service	Blended learning	Both	Facility and classroom	Tablet and mobile phone	Videos	Online	Not reported	Not reported	7 weeks	Not reported	Learning, group discussion, and feedback
Khan et al [37]	Preservice	Digital training	Asynchronous	Community	Tablet	Videos	Haiti: online and offline; India: offline	Yes	Not reported	Haiti: 8 months ; India: 4 months	Haiti: 39 modules; India: 14 modules	Learning, group discussion, and practice
Kharel et al [38]	In-service	Digital training	Synchronous	Community	Not reported	Videos	Online	No	4 hours	1 day	3 modules	Learning, modeling, and discussion
Lakshminarayanan et al [39]	In-service	Digital training	Both	Community	Mobile phone, tablets, and basic computers	Videos	Online	no	20‐25 h	1 month	10 sessions	Learning
Limaye et al [40]	In-service	Digital training	Asynchronous	Community	Computer	Videos	Offline	Yes	Not reported	4 months	8 courses	Learning
Muke et al [41]	In-service	Digital training	Synchronous	Conference or classroom	Tablets, mobile phone, and laptops	Videos	Online	No	2‐3 hours	1 day	2 modules	Learning
Muke et al [42]	In-service	Digital training	Synchronous	Community	Mobile phone	Videos	Online	No	48 hours	30 days	16 modules	Learning and modeling
Nedungadi et al [43]	In-service	Digital training	Synchronous	Conference	Computer	Videos	Online	No	Not reported	1 day	Not reported	Learning and practicing
O’Donovan et al [44]	In-service	Digital training	Asynchronous	Community	Tablet	Videos	Offline	Yes	Not reported	5 days	4 sessions	Learning
Rahman et al [45]	In-service	Digital training	Both	Community	Tablet	Videos	Offline	No	20 hours	5 days	Not reported	Learning, modeling, and group discussion
Sangwa et al [46]	In-service	Digital training	Asynchronous	Community	Mobile phone	Videos	Online	No	Not reported	4 weeks	4 sessions	Learning
Sranacharoenpong et al [47]	In-service	Blended learning	Both	Conference and community	Computer	Videos	Online and offline	No	20‐24 h	4 months	8 modules	Learning and group discussion
Sranacharoenpong and Hanning [48]	In-service	Blended learning	Both	Conference and community	Computer	Videos	Online and offline	No	Not reported	4 months	8 modules	Learning and group discussion
Tembo et al [49]	In-service	Blended learning	Synchronous	Conference	Computer	Videos	Offline	Yes	16 hours	2 days	Not reported	Learning, modeling, practicing, and feedback
Willems et al [50]	In-service	Digital training	Asynchronous	Community	Feature phone	Audios	Offline	Yes	40 minutes	4 weeks	8 modules	Learning
Yadav et al [51]	In-service	Digital training	Synchronous	Community	Mobile phone and feature phone	Audios	Online and offline	No	18 hours	22 days	12 sessions	Learning and group discussion
Yadav [52]	In-service	Digital training	Synchronous	Community	Mobile phone and feature phone	Audios	Online and offline	No	18 hours	22 days	12 sessions	Learning and group discussion
Yadav et al [53]	In-service	Digital training	synchronous	Community	Mobile phone and feature phone	Audios	Online	No	7.5‐10 h	6 weeks	10 sessions	Group discussion

**Table 3.** Panel of the population, training description, and outcomes.

Studies	Training description and outcomes
Bertman et al [36]	Who: a total of 293 primary counselors from 233 health facilities in Zimbabwe were trained in the Ministry of Health HIV testing guidelines for children and adolescents. What: the 7-week blended training included tablet-based self-study, a one-and-a-half-day classroom session at the beginning of the course, and a 4-hour classroom session at the end. The self-study component included videos, podcasts, case studies, quizzes, and self-reflection questions. These activities were complemented by the SMS text messaging component, including a whole-group discussion forum as well as 2-person partner discussions. The course was facilitated by 3 trainers who presented content, managed logistics, and interacted with the WhatsApp (Meta) groups. The training incorporated group discussions and peer support through WhatsApp, where participants could share complex cases, seek advice, and engage in problem-solving. Outcome: the SMS text messaging–based peer support and learning platform was well-received by participants, with high levels of engagement in case discussions while ensuring anonymity. Feedback indicated that the WhatsApp groups helped identify knowledge gaps and provided meaningful learning opportunities. The platform facilitated access to supervisors and experts, enriching the learning experience and fostering collaboration.
Khan et al [37]	Who: a total of 6 previously untrained lay individuals in Haiti and 55 from India were trained as acute care providers (ACP) for general acute medical conditions. What: trainees participated in electronic, asynchronous training using videos preloaded on tablets focusing on the diagnosis, treatment, and triage of 30 acute care chief complaints specific to these communities. The training included self-study modules, weekly discussions via video conferences, and multiple assessments. In Haiti, trainees completed 39 modules over 8 months, while in India, they completed 14 modules over 4 months. Outcome: ACP demonstrated significant improvements in knowledge in diagnosing acute conditions. The training program was feasible to implement, with nearly all completing the training and passing the posttest.
Kharel et al [38]	Who: a total of 183 female community health volunteers (FCHVs) in rural Nepal were trained to support the COVID-19 response. What: the 3-module digital training program aimed to equip FCHVs with practical knowledge and skills to combat the COVID-19 pandemic. The 4-hour training included interactive didactic modules, case-based learning, discussions, and video simulations. Training materials were translated into Nepali and delivered by a lead facilitator fluent in the language. Outcome: the training led to significant improvements in knowledge among FCHVs. The program demonstrated that virtual training is feasible in low-resource settings and can effectively enhance the capacity of community health workers.
Lakshminarayanan et al [39]	Who: a total of 23 lay counselors were trained to understand, identify, and respond to perinatal mental health services in India. What: the digital training had 10 sessions delivered over a period of 1 month, each session lasted 2-2.5 h. Training was delivered via videos, videoconferencing and instant messaging, file transfer, screen sharing via mobile phones, tablets, and basic computers. Outcome: trained counselors were satisfied and improved knowledge to deliver mental health counseling.
Limaye et al [40]	Who: a total of 306 frontline workers (FWs) in rural Bangladesh were trained to promote family planning and maternal, newborn, and child health. What: a digital training package was implemented for a 4-month duration using 8 courses accessible offline via netbooks (basic computers). Training incorporated multimedia elements, interactive learning, videos, and text messages to reinforce key concepts. Outcome: significant improvements in mean knowledge scores across family planning and maternal, newborn, and child health topics were observed. Measurable changes in counseling behaviors were reported.
Muke et al [41]	Who: a total of 32 accredited social health activists (ASHAs) in rural India were trained to treat depression by delivering the Healthy Activity Program (HAP). What: training was conducted using videos, PowerPoints (Microsoft Corp) with voice-over narration using tablets, mobile phones, and laptops. Training was for 2‐3 h. Outcome: ASHAs found the training acceptable for learning new content and feasible to use.
Muke et al [42]	Who: a total of 45 ASHAs participated in the study in Madhya Pradesh, India, and were trained to treat depression by delivering the Healthy Activity Program (HAP). What: ASHAs randomized to the digital training received 48 hours of training over 30 days using a mobile phone through the Moodle Learning Management System. The training sessions consisted of videos, role-play videos, PowerPoint presentations, and reading materials. Outcome: the training improved knowledge and skills in delivering the HAP for depression.
Nedungadi et al [43]	Who: a total of 23 community health workers (CHWs) trained in the management of communicable and noncommunicable diseases and nutritional deficiency in rural India. What: the SwastyaSIM is a digital training accessible online via a smartphone or computer designed to improve health literacy among CHWs by offering interactive simulations for medical training, including diagnostic tests. Outcome: SwastyaSIM significantly improved the knowledge of CHWs, particularly in infection control practices.
O’Donovan et al [44]	Who: a total of 163 community health workers in Mukono, Uganda, participated in a pilot randomized controlled trial. What: CHWs participated in a digital training to recognize, treat, and prevent pneumonia in accordance with integrated Community Case Management (iCCM) using tablets preloaded with videos in the local dialect (Luganda) with English subtitles for 5 days. Outcome: training showed improvements in test scores, in terms of knowledge acquisition post training.
Rahman et al [45]	Who: a total of 80 lady health workers (LHWs) in Pakistan were trained in the psychosocial management of perinatal depression. What: LHWs participated in the 20-hour digital training consisting of videos, role-play videos, sharing experiences, and problem-solving strategies over 5 days. Outcome: LHW competency improved after the training.
Sangwa et al [46]	Who: a total of 36 CHWs in Rutsiro (rural) and Ngoma (periurban) districts of Rwanda were trained in maternal and neonatal health. What: the training was delivered via the Ministry of Health’s (MOH) eLearning platform, based on Moodle (Moodle Pty Ltd) through a smartphone over 4 weeks. Outcome: postintervention and 6-month follow-up tests showed a significant increase in knowledge. Older CHWs performed better in follow-up tests, while all experience levels demonstrated consistent improvement, highlighting the effectiveness of eLearning across diverse learner groups.
Sranacharoenpong et al [47]	Who: a total of 69 community health care workers (CHCWs) in Thailand were trained in diabetes prevention education training. What: the training integrated traditional and e-learning approaches, using 8 in-classroom sessions and 8 online learning sessions for a 4-month duration. Each session was for 2.5‐3 h. Learning materials included videos, lecture notes, PowerPoint presentations, and monthly newsletters. A facilitator coordinated discussions and provided virtual support during the training period. Outcome: postintervention knowledge scores significantly improved. Participants demonstrated enhanced skills in measuring health metrics and understanding dietary recommendations.
Sranacharoenpong and Hanning [48]	Who: a total of 69 community health care workers (CHCWs) were trained in a tailored diabetes prevention education program in Chiang Mai, Thailand. What: the intervention involved 8 group classes and 8 self-directed e-learning sessions using videos via a computer over 4 months. Each session was for 2.5-3 h. Outcome: knowledge improvement was significant, and CHCWs felt confident in applying the knowledge and believed they could teach at-risk populations about diabetes prevention.
Tembo et al [49]	Who: a total of 12 health care workers (HCWs) from 6 facilities in Southern Malawi were trained to deliver Malawi’s index case testing services. What: the HCWs were trained using a blended learning package (combining digital and face-to-face approaches) for 16 hours in 2 days to improve Malawi’s HIV index case testing services. Outcome: the blended learning package resulted in significant improvements in HCWs’ fidelity to the index testing protocol. The package was perceived to be acceptable by HCWs and improved a number of meaningful clinical indicators, including index clients counseled, contacts elicited, and contacts who reported for HIV testing.
Willems et al [50]	Who: over 51,000 CHWs across Rwanda participated in a nationwide remote training service (RTS) focused on mental health education. What: the RTS used Interactive Voice Response (IVR) technology to deliver audio-based training modules to CHWs via simple feature phones. Over 4 weeks, 2 modules lasting 5 minutes each were released weekly, totaling 8 modules covering topics such as common mental disorders, posttraumatic stress disorder (PTSD), depression, drug abuse, and epilepsy. Outcome: knowledge of mental health topics significantly improved after the training. CHWs’ awareness and self-confidence in identifying and referring patients with mental health conditions were enhanced.
Yadav et al [51]	Who: a total of 40 ASHAs in India participated in a maternal and child health care training. What: the digital training included 12 sessions covering 10 topics over a period of 22 days. The content was adapted to local contexts and recorded in an audio dialog format. ASHAs accessed training via feature phones, while trainers used smartphones with internet connectivity to conduct the training. Outcome: the ASHAs showed a significant improvement in knowledge postintervention compared to the control group.
Yadav [52]	Who: a total of 40 community health workers in India participated in the training program. What: the digital training consists of facilitators using a smartphone scheduling to call participants through their feature phones. The facilitator delivered the training using audios and needed an internet connection to conduct the training while the participant needs to be where there is a telecommunication network to participate in the training. Outcome: the training program significantly improved ASHAs’ knowledge on maternal and child health. The training allowed for interaction and discussion with experts and peer learning, fostering active engagement.
Yadav et al [53]	Who: a total of 120 ASHAs in India were trained in maternal and child health through a peer-led educational intervention. What: ASHAs were provided with prerecorded audio learning materials in the native language (Hindi) and were expected to listen to 3 sessions via feature phones and discuss as a small group discussion. The duration of a single session was 15 minutes. Sessions were scheduled in the afternoons with flexibility to reschedule. Group facilitators (ASHA with a smartphone) played a key role in leading discussions and promoting engagement. Outcome: the intervention resulted in significant knowledge gains and ASHAs reported feeling empowered through peer discussions and knowledge sharing.

### Training Outcomes

Fourteen studies reported learning outcomes (Table 4). The learning outcomes reported in the studies were knowledge, attitude, and behaviors. Assessment of knowledge was made mainly through pretraining and posttraining tests (n=13) [37-40,42,44-48,50-53]. All 13 studies reported improved knowledge outcomes after the training. Two studies reported improved attitude. One study from India that implemented both synchronous and asynchronous learning approaches reported enhanced attitude (confidence) in delivering maternal mental health services [39] and another from Rwanda demonstrated asynchronous learning improvements in providing support to patients with mental health conditions in the community [50]. One study from Bangladesh reported that asynchronous learning improved behavior in counseling couples on all available contraceptive options and child spacing [40].

The reported outcomes varied across the studies. Most studies presented one outcome from one category. Table 5 summarizes the available literature, notes gaps of digital training outcomes where little or no research has been conducted, and highlights areas for future research. Ten studies reported implementation outcomes [36,37,41-44,49-53]. Seven studies used synchronous learning [41-43,49,51-53], 3 asynchronous learning [37,44,50] and 2 studies implemented both approaches [36,39]. The implementation outcomes reported were acceptability, appropriateness, feasibility, and fidelity. Acceptability included perceived effectiveness, perception of remote training, usability, and satisfaction (n=10) [36,39,41-44,49-52]. Acceptability was assessed posttraining mainly through questionnaires, written qualitative feedback, or in-depth interviews. Three studies reported that participants were satisfied with the training [39,47,50] and the other 7 reported that the training was agreeable. Two studies reported on appropriateness and found the training suitable for capacity building for CHWs [41,50].

**Table 4.** Digital and blended learning training, implementation, and clinical outcomes.

Study	Analytic approach	Knowledge	Attitude	Behaviors	Acceptability	Appropriateness	Feasibility	Fidelity	Effectiveness
Bertman et al [36]	Qualitative	No	No	No	Yes	No	No	No	No
Khan et al [37]	Quantitative	Yes	No	No	No	No	Yes	No	No
Kharel et al [38]	Quantitative	Yes	No	No	No	No	No	No	No
Lakshminarayanan et al [39]	Quantitative	Yes	Somewhat	No	Somewhat	No	Somewhat	No	No
Limaye et al [40]	Quantitative	Yes	No	Yes	No	No	No	No	No
Muke et al [41]	Qualitative	No	No	No	Yes	Yes	Yes	No	No
Muke et al [42]	Mixed	Yes	No	No	Yes	No	Yes	No	No
Nedungadi et al [43]	Mixed	No	No	No	Yes	No	No	No	No
O’Donovan et al [44]	Quantitative	Yes	No	No	Yes	No	No	No	No
Rahman et al [45]	Quantitative	Yes	No	No	No	No	No	No	No
Sangwa et al [46]	Quantitative	Yes	No	No	No	No	No	No	No
Sranacharoenpong et al [47]	Quantitative	Yes	No	No	No	No	No	No	No
Sranacharoenpong and Hanning [48]	Quantitative	Yes	No	No	No	No	No	No	No
Tembo et al [49]	Quantitative	No	No	No	Yes	No	No	Yes	Yes
Willems et al [50]	Mixed	Yes	Yes	No	Yes	Yes	No	Yes	No
Yadav et al [51]	Mixed	Yes	No	No	Yes	No	Yes	No	No
Yadav [52]	Mixed	Yes	No	No	Yes	No	Yes	No	No
Yadav et al [53]	Mixed	Yes	No	No	No	No	Yes	No	No

Less than half of the studies reported on the feasibility of participating in digital training, and all found the training was successfully carried out (feasible) [37,39,41,42,51-53]. Feasibility assessment mostly involved posttraining surveys or qualitative interviews or feedback or reviewing training logs. This included responses to questions about digital device familiarity [41] to the review of log-in attempts and the number of hours spent in the training system [51]. Some studies reported technical challenges that affected training feasibility. Three studies reported poor internet connectivity challenges, including lag or slow internet [38,41,42]. Four studies reported poor cellular network infrastructure [36,51-53]. One mentioned other technical glitches [39] and one discussed power cuts hindering charging tablets [36].

One study from Rwanda reported high fidelity to the asynchronous training schedule [50]. Another study from Malawi found synchronous learning improved fidelity in providing counseling services to patients as intended based on a CHW checklist [49]. This study also reported improved effectiveness on index case testing indicators, such as sexual contacts elicited [49]. This was the only clinical outcome. Full details of the outcomes for individual studies are summarized in Table 3.

**Table 5.** Training outcomes of the included studies.

Study	Learning outcomes			Implementation outcomes							Clinical outcomes				
	Knowledge	Attitudes	Skills	Behavior	Acceptability	Feasibility	Fidelity	Appropriateness	Sustainability	Uptake	Effectiveness	Efficiency	Safety	Equity	Patient satisfaction
Bertman et al [36]					✓										
Sranacharoenpong et al [47]	✓														
Sranacharoenpong and Hanning [48]	✓														
Tembo et al [49]					✓		✓				✓				
Khan et al [37]	✓					✓									
Kharel et al [38]	✓														
Lakshminarayanan et al [39][39]	✓														
Limaye et al [40]	✓			✓											
Muke et al [41]					✓	✓		✓							
Muke et al [42][42]	✓				✓	✓									
Nedungadi et al [43]					✓										
O’Donovan et al [44]	✓				✓										
Rahman et al [45]	✓														
Sangwa et al [46]	✓														
Willems et al [50]	✓	✓			✓	✓	✓								
Yadav et al [51]	✓				✓	✓									
Yadav [52]	✓				✓	✓									
Yadav et al [53]	✓					✓									

## Discussion

### Principal Findings

This scoping review brings together studies and reports of the characteristics and outcomes of digital training for CHWs in LMICs. Despite a rigorous search across multiple databases and broad inclusion criteria, we identified only 18 studies, which highlights the dearth of robust evaluations of digital training in LMICs. Yet, the review indicated that despite technological and infrastructural challenges, training CHWs using digital technologies was acceptable and feasible and led to knowledge acquisition. This body of evidence was largely descriptive, with few studies using experimental or quasi-experimental designs. Most reported only on learning outcomes, with very few reporting on implementation fidelity or clinical outcomes as a result of CHW training. Additionally, the majority of studies in this review only measured Kirkpatrick [25] Levels 1 and 2. A few measured Level 3 behaviors, and only one measured Level 4 results. This highlights the need for additional research evaluating additional level 3 and 4 outcomes.

This is the first review of digital training among CHWs in LMIC. Other reviews have focused on digital training primarily for professional health workers, mostly from HICs [15,16]. These reviews have similarly found relatively few studies, mostly descriptive in nature, with only knowledge outcomes evaluated. A scoping review that focused on digital training for rural professional cadres [16] identified few nonexperimental studies (n=5) and focused mainly on knowledge acquisition. Similarly, a systematic review that evaluated digital learning for medical education in LMICs [54] found two-thirds of the studies identified were from upper-middle-income countries, and only 4 were RCTs. Additionally, a review of digital learning for professional HCWs [15] found a few studies (n=14) conducted in LMICs; only 7 were RCTs and mainly reported knowledge outcomes.

Most of the evidence gathered in our scoping review showed that the digital or blended learning training resulted in significantly improved knowledge of CHWs after the training, compared to before, and is consistent with other learning modalities [55,56]. Similarly, our results are consistent with other studies in medical education reporting on the effectiveness of digital or blended learning in enhancing knowledge acquisition among certified health professionals in both LMICs [57,58] and HICs [59-61]. This supports the finding that digital training accounts for the knowledge needed to perform tasks across disease entities and health cadres in both high- and lower-economic settings. Furthermore, a systematic review comparing the effectiveness of blended learning to traditional learning for professional or certified HCWs [17] concluded that blended learning demonstrated consistently positive effects on knowledge outcomes when compared with traditional learning in medical education. Our work extends these findings to CHWs’ knowledge to perform delegated assignments.

A few studies included in this scoping review addressed acceptability and appropriateness [41,50]. Feedback from CHWs suggested that they found digital or blended training acceptable for learning new information and appropriate as a mode of training. This has also been reported in other studies where digital training was acceptable among CHWs in diverse settings for data collection training [62]. Acceptability and appropriateness are important factors for training completion and skills application.

Overall, digital training was feasible, though approaches differed considerably. This is consistent with other studies with professional cadres [15,63]. For example, studies in Kenya [14] and Nigeria [57] found digital training for HCWs was feasible at scale. These studies highlight the potential feasibility of implementing digital training interventions in LMICs. Digital training programs in this review included the use of various technologies, including tablets, mobile phones, or feature phones. Some training approaches did not require internet or physical infrastructure, which may be lacking or unreliable in low-resource settings. For example, CHWs could access the training on a feature phone in the community without needing internet access or being in centralized locations [50]. The use of mobile devices made it possible for synchronous or asynchronous learning models to occur. Both models appear to be of benefit. Asynchronous learning offered CHWs the opportunity to participate in the training in a flexible manner at a time and place that suited them without missing any material due to other demanding personal or work-related responsibilities. Synchronous learning offered CHWs the opportunity to participate in group discussions, ask questions in real-time, and practice and receive feedback. Furthermore, synchronous sessions provided the trainees with access to technical support periodically. With the increased use of smartphones in LMICs, digital training could successfully be implemented in LMICs [64].

This review also underlined that using digital training programs in LMICs will require technological challenges to be overcome. Technical challenges, including slow or poor internet and cellular networks, were frequently cited as barriers to training completion. For example, Muke et al [42] reported that due to the challenge of poor internet connectivity, some participants were unable to access all content from the training modules because videos did not play. Preloading training material could eliminate reliance on connectivity and may ensure availability offline [65]. In addition, using feature phones demonstrated the possibility of mitigating network challenges [50]. The design of the digital training program is imperative. Simpler platforms may be easier to use and scale, and good accessibility may provide efficiencies that enable greater impact of the training.

### Policy and Practice Recommendations

Given the global shortage of professional health workers, recruiting and training CHWs is one solution to overcome this gap in LMICs. The integration of digital training programs into national health strategies could play a pivotal role in achieving high-quality health care outcomes. LMICs should consider investing in digital infrastructure, including technology devices and literacy for lay cadres, to enhance accessibility to training and scalability of digital solutions. Tailoring and co-designing digital training programs with CHWs to ensure relevance and practicality will encourage engagement and sustainability of the digital tools. These recommendations support the WHO’s suggestion of using digital training to supplement health workers’ continuous capacity-building efforts [66].

### Limitations

Our review had important limitations. Some studies did not provide enough details on training characteristics and outcomes. There was a potential to give greater emphasis to the studies that provided more detailed information when using narrative synthesis in data analysis. To overcome this, we contacted all authors for more information and were able to obtain some additional data that were not published in the articles. Additionally, we extracted data according to a predesigned structure and had 2 authors (TAT and FA) extract data independently.

### Conclusion

CHWs play an essential role in providing health care and improving health outcomes in LMIC environments. Training must continue to be a core component of improving CHW skills. In LMIC environments that often have lower technological literacy and infrastructure, we sought to understand what role digital training could play. Digital trainings had a broad range of attributes. Few evaluations of digital training for CHWs in LMICs were identified in this review, in spite of a proliferation of such trainings. Furthermore, most evaluations lacked experimental designs. The existing evidence suggests that digital training can impact knowledge in lay health workers in LMICs; more rigorous studies with a broader range of outcomes are needed. Such additional work is needed to ensure lay health workers are well-capacitated to deliver critical health services.

## Supplementary material

10.2196/82772 Multimedia Appendix 1 Database search terms and results.

10.2196/82772 Checklist 1 PRISMA checklist.

## Abbreviations

CHW: community health worker
CINAHL: Cumulated Index in Nursing and Allied Health Literature
ERIC: Education Resources Information Center
HCW: health care worker
HIC: high-income country
LMIC: low- and middle-income country
PRISMA: Preferred Reporting Items for Systematic Reviews and Meta-Analyses
PRISMA-S: Preferred Reporting Items for Systematic Reviews and Meta-Analyses–Search Extension
PRISMA-ScR: Preferred Reporting Items for Systematic Reviews and Meta-Analyses extension for Scoping Reviews
PROSPERO: International Prospective Register of Systematic Reviews
RCT: randomized controlled trial
SSRN: Social Science Research Network
WHO: World Health Organization
