# Supplementary material for: Outcomes of Digital Training for Community Health Workers in Low- and Middle-Income Countries: Scoping Review
Source: JMIR Med Educ. 2026 May 19;12:e82772. doi: 10.2196/82772 (PMC13186524; doi:10.2196/82772)
Supplement: Multimedia Appendix 1 [file mededu-v12-e82772-s001.docx]

**Table S1: Database search strategies, including search terms**

| Database(s) | Search strategy |
| --- | --- |
| PubMed | #4 - #1 AND #2 AND #3  #3 - Search: ((((((((((((((((((((((((((((((((((((((((((((((((((((((((((((((((((((((((((((((((((((((((((((((((((((((((((((((((((((((((((((((((((((((((((((((((((((((((((((((((((((((((((((((((((((((((((((((((((((((((((((((((developing countr*[Title/Abstract]) OR (developing countries[Title/Abstract])) OR (developing nation*[Title/Abstract])) OR (developing world[Title/Abstract])) OR (less-developed countr*[Title/Abstract])) OR (less developed countr*[Title/Abstract])) OR (less-developed world[Title/Abstract])) OR (lesser developed world[Title/Abstract])) OR (lesser-developed countr*[Title/Abstract])) OR (lesser developed countr*[Title/Abstract])) OR (lesser-developed nation*[Title/Abstract])) OR (lesser developed nation*[Title/Abstract])) OR (lesser-developed world[Title/Abstract])) OR (under-developed countr*[Title/Abstract])) OR (under developed countr*[Title/Abstract])) OR (under-developed nation*[Title/Abstract])) OR (under developed nation*[Title/Abstract])) OR (under-developed world[Title/Abstract])) OR (underdeveloped world[Title/Abstract])) OR (under developed world[Title/Abstract])) OR (underdeveloped countr*[Title/Abstract])) OR (under-developed countr*[Title/Abstract])) OR (Under developed countr*[Title/Abstract])) OR (under developed nation*[Title/Abstract])) OR (underdeveloped nation*[Title/Abstract])) OR (lower middle income country[Title/Abstract])) OR (lower middle-income country[Title/Abstract])) OR (lower middle income nation[Title/Abstract])) OR (lower middle income nation[Title/Abstract])) OR (upper middle-income country[Title/Abstract])) OR (upper middle income country[Title/Abstract])) OR (upper middle-income nation[Title/Abstract])) OR (upper middle income nation[Title/Abstract])) OR (low-income country[Title/Abstract])) OR (low income country[Title/Abstract])) OR (low-income nation[Title/Abstract])) OR (low income nation[Title/Abstract])) OR (lower income country[Title/Abstract])) OR (lower-income country[Title/Abstract])) OR (lower income nation[Title/Abstract])) OR (lower-income nation[Title/Abstract])) OR (lower middle income countries[Title/Abstract])) OR (lower middle-income countries[Title/Abstract])) OR (lower middle income nation[Title/Abstract])) OR (lower middle-income nation[Title/Abstract])) OR (upper middle-income countries[Title/Abstract])) OR (upper middle income countries[Title/Abstract])) OR (upper middle-income nation[Title/Abstract])) OR (upper middle income nation[Title/Abstract])) OR (low income countries[Title/Abstract])) OR (low income countries[Title/Abstract])) OR (low-income nation[Title/Abstract])) OR (low income nation[Title/Abstract])) OR (lower income countries[Title/Abstract])) OR (lower-income countries[Title/Abstract])) OR (lower income nation[Title/Abstract])) OR (lower-income nation[Title/Abstract])) OR (lmic[Title/Abstract])) OR (lmics[Title/Abstract])) OR (third world[Title/Abstract])) OR (lami countr*[Title/Abstract])) OR (transitional countr*[Title/Abstract])) OR (majority world[Title/Abstract])) OR (Global South[Title/Abstract])) OR (LMIC[Title/Abstract])) OR (LMICs[Title/Abstract])) OR (LIC[Title/Abstract])) OR (LICs[Title/Abstract])) OR (LMICs[Title/Abstract])) OR (LMIC[Title/Abstract])) OR (UMICs[Title/Abstract])) OR (UMIC[Title/Abstract])) OR (Afghanistan[Title/Abstract])) OR (Burkina Faso[Title/Abstract])) OR (Burundi[Title/Abstract])) OR (Central African Republic[Title/Abstract])) OR (Chad[Title/Abstract])) OR (Congo, Dem.[Title/Abstract])) OR (Eritrea[Title/Abstract])) OR (Ethiopia[Title/Abstract])) OR (Gambia[Title/Abstract])) OR (Guinea-Bissau[Title/Abstract])) OR (Korea, Dem. People's Rep[Title/Abstract])) OR (Liberia[Title/Abstract])) OR (Madagascar[Title/Abstract])) OR (Malawi[Title/Abstract])) OR (Mali[Title/Abstract])) OR (Mozambique[Title/Abstract])) OR (Niger[Title/Abstract])) OR (Rwanda[Title/Abstract])) OR (Sierra Leone[Title/Abstract])) OR (Somalia[Title/Abstract])) OR (South Sudan[Title/Abstract])) OR (Sudan[Title/Abstract])) OR (Syrian Arab Republic[Title/Abstract])) OR (Togo[Title/Abstract])) OR (Uganda[Title/Abstract])) OR (Yemen, Rep.[Title/Abstract])) OR (Angola[Title/Abstract])) OR (Algeria[Title/Abstract])) OR (Bangladesh[Title/Abstract])) OR (Benin[Title/Abstract])) OR (Bhutan[Title/Abstract])) OR (Bolivia[Title/Abstract])) OR (Cabo Verde[Title/Abstract])) OR (Cambodia[Title/Abstract])) OR (Cameroon[Title/Abstract])) OR (Comoros[Title/Abstract])) OR (Congo, Rep.[Title/Abstract])) OR (Côte d'Ivoire[Title/Abstract])) OR (Djibouti[Title/Abstract])) OR (Egypt, Arab Rep.[Title/Abstract])) OR (Eswatini[Title/Abstract])) OR (Ghana[Title/Abstract])) OR (Guinea[Title/Abstract])) OR (Haiti[Title/Abstract])) OR (Honduras[Title/Abstract])) OR (Jordan[Title/Abstract])) OR (India[Title/Abstract])) OR (Iran, Islamic Rep[Title/Abstract])) OR (Kenya[Title/Abstract])) OR (Kiribati[Title/Abstract])) OR (Kyrgyz Republic[Title/Abstract])) OR (Lao PDR[Title/Abstract])) OR (Lebanon[Title/Abstract])) OR (Lesotho[Title/Abstract])) OR (Mauritania[Title/Abstract])) OR (Micronesia, Fed. Sts.[Title/Abstract])) OR (Mongolia[Title/Abstract])) OR (Morocco[Title/Abstract])) OR (Myanmar[Title/Abstract])) OR (Nepal[Title/Abstract])) OR (Nicaragua[Title/Abstract])) OR (Nigeria[Title/Abstract])) OR (Pakistan[Title/Abstract])) OR (Papua New Guinea[Title/Abstract])) OR (Philippines[Title/Abstract])) OR (Samoa[Title/Abstract])) OR (São Tomé[Title/Abstract])) OR (Principe[Title/Abstract])) OR (Senegal[Title/Abstract])) OR (Solomon Islands[Title/Abstract])) OR (Sri Lanka[Title/Abstract])) OR (Tanzania[Title/Abstract])) OR (Tajikistan[Title/Abstract])) OR (Timor-Leste[Title/Abstract])) OR (Tunisia[Title/Abstract])) OR (Ukraine[Title/Abstract])) OR (Uzbekistan[Title/Abstract])) OR (Vanuatu[Title/Abstract])) OR (Vietnam[Title/Abstract])) OR (Zambia[Title/Abstract])) OR (Zimbabwe[Title/Abstract])) OR (Albania[Title/Abstract])) OR (Argentina[Title/Abstract])) OR (Armenia[Title/Abstract])) OR (Azerbaijan[Title/Abstract])) OR (Belarus[Title/Abstract])) OR (Belize[Title/Abstract])) OR (Bosnia[Title/Abstract])) OR (Herzegovina[Title/Abstract])) OR (Botswana[Title/Abstract])) OR (Brazil[Title/Abstract])) OR (Bulgaria[Title/Abstract])) OR (China[Title/Abstract])) OR (Colombia[Title/Abstract])) OR (Costa Rica[Title/Abstract])) OR (Cuba[Title/Abstract])) OR (Dominica[Title/Abstract])) OR (Dominican Republic[Title/Abstract])) OR (El Salvador[Title/Abstract])) OR (Equatorial Guinea[Title/Abstract])) OR (Ecuador[Title/Abstract])) OR (Fiji[Title/Abstract])) OR (Gabon[Title/Abstract])) OR (Georgia[Title/Abstract])) OR (Grenada[Title/Abstract])) OR (Guatemala[Title/Abstract])) OR (Indonesia[Title/Abstract])) OR (Iraq[Title/Abstract])) OR (Jamaica[Title/Abstract])) OR (Kazakhstan[Title/Abstract])) OR (Kosovo[Title/Abstract])) OR (Libya[Title/Abstract])) OR (Malaysia[Title/Abstract])) OR (Maldives[Title/Abstract])) OR (Marshall Islands[Title/Abstract])) OR (Mauritius[Title/Abstract])) OR (Mexico[Title/Abstract])) OR (Moldova[Title/Abstract])) OR (Montenegro[Title/Abstract])) OR (Namibia[Title/Abstract])) OR (North Macedonia[Title/Abstract])) OR (Palau[Title/Abstract])) OR (Paraguay[Title/Abstract])) OR (Peru[Title/Abstract])) OR (Russian Federation[Title/Abstract])) OR (Serbia[Title/Abstract])) OR (South Africa[Title/Abstract])) OR (St. Lucia[Title/Abstract])) OR (St. Vincent[Title/Abstract])) OR (Suriname[Title/Abstract])) OR (Thailand[Title/Abstract])) OR (Tonga[Title/Abstract])) OR (Türkiye[Title/Abstract])) OR (Turkmenistan[Title/Abstract])) OR (Tuvalu[Title/Abstract])) OR (West Bank[Title/Abstract])) OR (Gaza[Title/Abstract])  #2 - Search: (((((((((((((((((((((Community health care worker*[Title/Abstract]) OR (CHW*[Title/Abstract])) OR (Community health worker*[Title/Abstract])) OR (Front line health worker*[Title/Abstract])) OR (Outreach worker*[Title/Abstract])) OR (Lay health worker*[Title/Abstract])) OR (Lay counsellor*[Title/Abstract])) OR (Health promoter*[Title/Abstract])) OR (Village health worker*[Title/Abstract])) OR (Volunteer health worker*[Title/Abstract])) OR (Community volunteer*[Title/Abstract])) OR (Village health volunteer*[Title/Abstract])) OR (Lady health worker*[Title/Abstract])) OR (Barangay health worker*[Title/Abstract])) OR (Outreach educator*[Title/Abstract])) OR (Shas*[Title/Abstract])) OR (Shebika[Title/Abstract])) OR (Shastho karmis[Title/Abstract])) OR (Village health helper*[Title/Abstract])) OR (Accredited Social Health Activist, ASHA[Title/Abstract])) OR (Family Health Worker*[Title/Abstract])) OR (Peer Educator*[Title/Abstract])  #1- Search: ((((((((((((((((((((((Virtual learning[Title/Abstract]) OR (e-learning[Title/Abstract])) OR (elearning[Title/Abstract])) OR (Electronic learning[Title/Abstract])) OR (Online learning[Title/Abstract])) OR (Distance learning[Title/Abstract])) OR (Massive Open Online Course[Title/Abstract])) OR (MOOC[Title/Abstract])) OR (Mobile learning[Title/Abstract])) OR (m-learning[Title/Abstract])) OR (mlearning[Title/Abstract])) OR (Webbased[Title/Abstract])) OR (Web-based[Title/Abstract])) OR (Offline learning[Title/Abstract])) OR (Offline distance learning[Title/Abstract])) OR (Technology enhanced learning[Title/Abstract])) OR (Digital innovations[Title/Abstract])) OR (Hybrid learning[Title/Abstract])) OR (Flipped learning[Title/Abstract])) OR (Blended education[Title/Abstract])) OR (Multi-model learning[Title/Abstract])) OR (Hyflex learning[Title/Abstract])) OR (Asynchronous learning[Title/Abstract]) |
| CINHAL | S4 – S1 AND S2 AND S3  S3 - (TI "developing countr*" OR AB "developing countr*" OR TI "developing countries" OR AB "developing countries" OR TI "developing nation*" OR AB "developing nation*" OR TI "developing world" OR AB "developing world" OR TI "less-developed countr*" OR AB "less-developed countr*" OR TI "less developed countr*" OR AB "less developed countr*" OR TI "less-developed world" OR AB "less-developed world" OR TI "lesser developed world" OR AB "lesser developed world" OR TI "lesser-developed countr*" OR AB "lesser-developed countr*" OR TI "lesser developed countr*" OR AB "lesser developed countr*" OR TI "lesser-developed nation*" OR AB "lesser-developed nation*" OR TI "lesser developed nation*" OR AB "lesser developed nation*" OR TI "lesser-developed world" OR AB "lesser-developed world" OR TI "under-developed countr*" OR AB "under-developed countr*" OR TI "under developed countr*" OR AB "under developed countr*" OR TI "under-developed nation*" OR AB "under-developed nation*" OR TI "under developed nation*" OR AB "under developed nation*" OR TI "under-developed world" OR AB "under-developed world" OR TI "underdeveloped world" OR AB "underdeveloped world" OR TI "under developed world" OR AB "under developed world" OR TI "underdeveloped countr*" OR AB "underdeveloped countr*" OR TI "under-developed countr*" OR AB "under-developed countr*" OR TI "under developed nation*" OR AB "under developed nation*" OR TI "underdeveloped nation*" OR AB "underdeveloped nation*" OR TI "lower middle income country" OR AB "lower middle income country" OR TI "lower middle-income country" OR AB "lower middle-income country" OR TI "lower middle income nation*" OR AB "lower middle income nation*" OR TI "lower middle-income nation*" OR AB "lower middle-income nation*" OR TI "upper middle-income country" OR AB "upper middle-income country" OR TI "upper middle income country" OR AB "upper middle income country" OR TI "upper middle-income nation*" OR AB "upper middle-income nation*" OR TI "upper middle income nation*" OR AB "upper middle income nation*" OR TI "low-income country" OR AB "low-income country" OR TI "low income country" OR AB "low income country" OR TI "low-income nation*" OR AB "low-income nation*" OR TI "low income nation*" OR AB "low income nation*" OR TI "lower income country" OR AB "lower income country" OR TI "lower-income country" OR AB "lower-income country" OR TI "lower income nation*" OR AB "lower income nation*" OR TI "lower-income nation*" OR AB "lower-income nation*" OR TI "lmic" OR AB "lmic" OR TI "lmics" OR AB "lmics" OR TI "third world" OR AB "third world" OR TI "lami countr*" OR AB "lami countr*" OR TI "transitional countr*" OR AB "transitional countr*" OR TI "majority world" OR AB "majority world" OR TI "global south" OR AB "global south" OR TI LMIC OR AB LMIC OR TI LMICs OR AB LMICs OR TI LIC OR AB LIC OR TI LICs OR AB LICs OR TI LMICs OR AB LMICs OR TI LMIC OR AB LMIC OR TI UMICs OR AB UMICs OR TI UMIC OR AB UMIC OR TI Afghanistan OR AB Afghanistan OR TI Burkina Faso OR AB Burkina Faso OR TI Burundi OR AB Burundi OR TI "Central African Republic" OR AB "Central African Republic" OR TI Chad OR AB Chad OR TI "Congo, Dem." OR AB "Congo, Dem." OR TI Eritrea OR AB Eritrea OR TI Ethiopia OR AB Ethiopia OR TI Gambia OR AB Gambia OR TI "Guinea-Bissau" OR AB "Guinea-Bissau" OR TI "Korea, Dem. People's Rep" OR AB "Korea, Dem. People's Rep" OR TI Liberia OR AB Liberia OR TI Madagascar OR AB Madagascar OR TI Malawi OR AB Malawi OR TI Mali OR AB Mali OR TI Mozambique OR AB Mozambique OR TI Niger OR AB Niger OR TI Rwanda OR AB Rwanda OR TI "Sierra Leone" OR AB "Sierra Leone" OR TI Somalia OR AB Somalia OR TI "South Sudan" OR AB "South Sudan" OR TI Sudan OR AB Sudan OR TI "Syrian Arab Republic" OR AB "Syrian Arab Republic" OR TI Togo OR AB Togo OR TI Uganda OR AB Uganda OR TI Yemen, Rep. OR AB "Yemen, Rep." OR TI Angola OR AB Angola OR TI Algeria OR AB Algeria OR TI Bangladesh OR AB Bangladesh OR TI Benin OR AB Benin OR TI Bhutan OR AB Bhutan OR TI Bolivia OR AB Bolivia OR TI "Cabo Verde" OR AB "Cabo Verde" OR TI Cambodia OR AB Cambodia OR TI Cameroon OR AB Cameroon OR TI Comoros OR AB Comoros OR TI "Congo, Rep." OR AB "Congo, Rep." OR TI "Côte d'Ivoire" OR AB "Côte d'Ivoire" OR TI Djibouti OR AB Djibouti OR TI "Egypt, Arab Rep." OR AB "Egypt, Arab Rep." OR TI Eswatini OR AB Eswatini OR TI Ghana OR AB Ghana OR TI Guinea OR AB Guinea OR TI Haiti OR AB Haiti OR TI Honduras OR AB Honduras OR TI Jordan OR AB Jordan OR TI India OR AB India OR TI "Iran, Islamic Rep" OR AB "Iran, Islamic Rep" OR TI Kenya OR AB Kenya OR TI Kiribati OR AB Kiribati OR TI "Kyrgyz Republic" OR AB "Kyrgyz Republic" OR TI "Lao PDR" OR AB "Lao PDR" OR TI Lebanon OR AB Lebanon OR TI Lesotho OR AB Lesotho OR TI Mauritania OR AB Mauritania OR TI "Micronesia, Fed. Sts." OR AB "Micronesia, Fed. Sts." OR TI Mongolia OR AB Mongolia OR TI Morocco OR AB Morocco OR TI Myanmar OR AB Myanmar OR TI Nepal OR AB Nepal OR TI Nicaragua OR AB Nicaragua OR TI Nigeria OR AB Nigeria OR TI Pakistan OR AB Pakistan OR TI "Papua New Guinea" OR AB "Papua New Guinea" OR TI Philippines OR AB Philippines OR TI Samoa OR AB Samoa OR TI "São Tomé" OR AB "São Tomé" OR TI "Principe" OR AB "Principe" OR TI Senegal OR AB Senegal OR TI "Solomon Islands" OR AB "Solomon Islands" OR TI "Sri Lanka" OR AB "Sri Lanka" OR TI Tanzania OR AB Tanzania OR TI Tajikistan OR AB Tajikistan OR TI "Timor-Leste" OR AB "Timor-Leste" OR TI Tunisia OR AB Tunisia OR TI Ukraine OR AB Ukraine OR TI Uzbekistan OR AB Uzbekistan OR TI Vanuatu OR AB Vanuatu OR TI Vietnam OR AB Vietnam OR TI Zambia OR AB Zambia OR TI Zimbabwe OR AB Zimbabwe OR TI Albania OR AB Albania OR TI Argentina OR AB Argentina OR TI Armenia OR AB Armenia OR TI Azerbaijan OR AB Azerbaijan OR TI Belarus OR AB Belarus OR TI Belize OR AB Belize OR TI Bosnia OR AB Bosnia OR TI Herzegovina OR AB Herzegovina OR TI Botswana OR AB Botswana OR TI Brazil OR AB Brazil OR TI Bulgaria OR AB Bulgaria OR TI China OR AB China OR TI Colombia OR AB Colombia OR TI "Costa Rica" OR AB "Costa Rica" OR TI Cuba OR AB Cuba OR TI Dominica OR AB Dominica OR TI "Dominican Republic" OR AB "Dominican Republic" OR TI "El Salvador" OR AB "El Salvador" OR TI "Equatorial Guinea" OR AB "Equatorial Guinea" OR TI Ecuador OR AB Ecuador OR TI Fiji OR AB Fiji OR TI Gabon OR AB Gabon OR TI Georgia OR AB Georgia OR TI Grenada OR AB Grenada OR TI Guatemala OR AB Guatemala OR TI Indonesia OR AB Indonesia OR TI Iraq OR AB Iraq OR TI Jamaica OR AB Jamaica OR TI Kazakhstan OR AB Kazakhstan OR TI Kosovo OR AB Kosovo OR TI Libya OR AB Libya OR TI Malaysia OR AB Malaysia OR TI Maldives OR AB Maldives OR TI "Marshall Islands" OR AB "Marshall Islands" OR TI Mauritius OR AB Mauritius OR TI Mexico OR AB Mexico OR TI Moldova OR AB Moldova OR TI Montenegro OR AB Montenegro OR TI Namibia OR AB Namibia OR TI "North Macedonia" OR AB "North Macedonia" OR TI Palau OR AB Palau OR TI Paraguay OR AB Paraguay OR TI Peru OR AB Peru OR TI "Russian Federation" OR AB "Russian Federation" OR TI Serbia OR AB Serbia OR TI "South Africa" OR AB "South Africa" OR TI "St. Lucia" OR AB "St. Lucia" OR TI "St. Vincent" OR AB "St. Vincent" OR TI Suriname OR AB Suriname OR TI Thailand OR AB Thailand OR TI Tonga OR AB Tonga OR TI Türkiye OR AB Türkiye OR TI "Turkmenistan" OR AB "Turkmenistan" OR TI Tuvalu OR AB Tuvalu OR TI "West Bank" OR AB "West Bank" OR TI Gaza OR AB Gaza)  S2 - (TI "community health care worker*" OR AB "community health care worker*" OR TI CHW OR AB CHW OR TI CHWs OR AB CHWs OR TI "community health worker*" OR AB "community health worker*" OR TI "front line health worker*" OR AB "front line health worker*" OR TI "outreach worker*" OR AB "outreach worker*" OR TI "lay health worker*" OR AB "lay health worker*" OR TI "lay counsellor*" OR AB "lay counsellor*" OR TI "health promoter*" OR AB "health promoter*" OR TI "village health worker*" OR AB "village health worker*" OR TI "volunteer health worker*" OR AB "volunteer health worker*" OR TI "community volunteer*" OR AB "community volunteer*" OR TI "village health volunteer*" OR AB "village health volunteer*" OR TI "lady health worker*" OR AB "lady health worker*" OR TI "barangay health worker*" OR AB "barangay health worker*" OR TI "outreach educator*" OR AB "outreach educator*" OR TI Shas* OR AB Shas* OR TI Shebika OR AB Shebika OR TI "shastho karmis" OR AB "shastho karmis" OR TI "village health helper*" OR AB "village health helper*" OR TI "accredited social health activist" OR AB "accredited social health activist" OR TI ASHA OR AB ASHA OR TI "family health worker*" OR AB "family health worker*" OR TI "peer educator*" OR AB "peer educator*")  S1 - (TI "virtual learning" OR AB "virtual learning" OR TI e-learning OR AB e-learning OR TI elearning OR AB elearning OR TI "electronic learning" OR AB "electronic learning" OR TI "online learning" OR AB "online learning" OR TI "distance learning" OR AB "distance learning" OR TI "massive open online course" OR AB "massive open online course" OR TI MOOC OR AB MOOC OR TI "mobile learning" OR AB "mobile learning" OR TI m-learning OR AB m-learning OR TI mlearning OR AB mlearning OR TI "webbased" OR AB "webbased" OR TI "web-based" OR AB "web-based" OR TI "offline learning" OR AB "offline learning" OR TI "offline distance learning" OR AB "offline distance learning" OR TI "technology enhanced learning" OR AB "technology enhanced learning" OR TI "digital innovations" OR AB "digital innovations" OR TI "hybrid learning" OR AB "hybrid learning" OR TI "flipped learning" OR AB "flipped learning" OR TI "blended education" OR AB "blended education" OR TI "multi-model learning" OR AB "multi-model learning" OR TI "hyflex learning" OR AB "hyflex learning" OR TI "asynchronous learning" OR AB "asynchronous learning") |
| SCOPUS | ( TITLE-ABS-KEY ( "virtual learning" OR "e-learning" OR "elearning" OR "electronic learning" OR "online learning" OR "distance learning" OR "massive open online course" OR "mooc" OR "mobile learning" OR "m-learning" OR "mlearning" OR "webbased" OR "web-based" OR "offline learning" OR "offline distance learning" OR "technology enhanced learning" OR "digital innovations" OR "hybrid learning" OR "flipped learning" OR "blended education" OR "multi-model learning" OR "hyflex learning" OR "asynchronous learning" ) ) AND ( TITLE-ABS-KEY ( "community health care worker*" OR "chw*" OR "community health worker*" OR "front line health worker*" OR "outreach worker*" OR "lay health worker*" OR "lay counsellor*" OR "health promoter*" OR "village health worker*" OR "volunteer health worker*" OR "community volunteer*" OR "village health volunteer*" OR "lady health worker*" OR "barangay health worker*" OR "outreach educator*" OR "shas*" OR "shebika" OR "shastho karmis" OR "village health helper*" OR "accredited social health activist, asha" OR "family health worker*" OR "peer educator*" ) ) AND ( TITLE-ABS-KEY ( "developing countr*" OR "developing countries" OR "developing nation*" OR "developing world" OR "less-developed countr*" OR "less developed countr*" OR "less-developed world" OR "lesser developed world" OR "lesser-developed countr*" OR "lesser developed countr*" OR "lesser-developed nation*" OR "lesser developed nation*" OR "lesser-developed world" OR "under-developed countr*" OR "under developed countr*" OR "under-developed nation*" OR "under developed nation*" OR "under-developed world" OR "underdeveloped world" OR "under developed world" OR "underdeveloped countr*" OR "under-developed countr*" OR "under developed countr*" OR "under developed nation*" OR "underdeveloped nation*" OR "lower middle income country" OR "lower middle-income country" OR "lower middle income nation" OR "lower middle income nation" OR "upper middle-income country" OR "upper middle income country" OR "upper middle-income nation" OR "upper middle income nation" OR "low-income country" OR "low income country" OR "low-income nation" OR "low income nation" OR "lower income country" OR "lower-income country" OR "lower income nation" OR "lower-income nation" OR "lower middle income countries" OR "lower middle-income countries" OR "lower middle income nation" OR "lower middle-income nation" OR "upper middle-income countries" OR "upper middle income countries" OR "upper middle-income nation" OR "upper middle income nation" OR "low income countries" OR "low income countries" OR "low-income nation" OR "low income nation" OR "lower income countries" OR "lower-income countries" OR "lower income nation" OR "lower-income nation" OR "lmic" OR "lmics" OR "third world" OR "lami countr*" OR "transitional countr*" OR "majority world" OR "global south" OR "lmic" OR "lmics" OR "lic" OR "lics" OR "lmics" OR "lmic" OR "umics" OR "umic" OR "afghanistan" OR "burkina faso" OR "burundi" OR "central african republic" OR "chad" OR "congo, dem." OR "eritrea" OR "ethiopia" OR "gambia" OR "guinea-bissau" OR "korea, dem. people&apos;s rep" OR "liberia" OR "madagascar" OR "malawi" OR "mali" OR "mozambique" OR "niger" OR "rwanda" OR "sierra leone" OR "somalia" OR "south sudan" OR "sudan" OR "syrian arab republic" OR "togo" OR "uganda" OR "yemen, rep." OR "angola" OR "algeria" OR "bangladesh" OR "benin" OR "bhutan" OR "bolivia" OR "cabo verde" OR "cambodia" OR "cameroon" OR "comoros" OR "congo, rep." OR "co&#770;te d&apos;ivoire" OR "djibouti" OR "egypt, arab rep." OR "eswatini" OR "ghana" OR "guinea" OR "haiti" OR "honduras" OR "jordan" OR "india" OR "iran, islamic rep" OR "kenya" OR "kiribati" OR "kyrgyz republic" OR "lao pdr" OR "lebanon" OR "lesotho" OR "mauritania" OR "micronesia, fed. sts." OR "mongolia" OR "morocco" OR "myanmar" OR "nepal" OR "nicaragua" OR "nigeria" OR "pakistan" OR "papua new guinea" OR "philippines" OR "samoa" OR "sa&#771;o tome&#769;" OR "principe" OR "senegal" OR "solomon islands" OR "sri lanka" OR "tanzania" OR "tajikistan" OR "timor-leste" OR "tunisia" OR "ukraine" OR "uzbekistan" OR "vanuatu" OR "vietnam" OR "zambia" OR "zimbabwe" OR "albania" OR "argentina" OR "armenia" OR "azerbaijan" OR "belarus" OR "belize" OR "bosnia" OR "herzegovina" OR "botswana" OR "brazil" OR "bulgaria" OR "china" OR "colombia" OR "costa rica" OR "cuba" OR "dominica" OR "dominican republic" OR "el salvador" OR "equatorial guinea" OR "ecuador" OR "fiji" OR "gabon" OR "georgia" OR "grenada" OR "guatemala" OR "indonesia" OR "iraq" OR "jamaica" OR "kazakhstan" OR "kosovo" OR "libya" OR "malaysia" OR "maldives" OR "marshall islands" OR "mauritius" OR "mexico" OR "moldova" OR "montenegro" OR "namibia" OR "north macedonia" OR "palau" OR "paraguay" OR "peru" OR "russian federation" OR "serbia" OR "south africa" OR "st. lucia" OR "st. vincent" OR "suriname" OR "thailand" OR "tonga" OR "tu&#776;rkiye" OR "turkmenistan" OR "tuvalu" OR "west bank" OR "gaza" ) ) |
| PsychINFO | S4 - S1 AND S2 AND S3  S3 - (TI(developing countr*) OR AB(developing countr*) OR TI(developing countries) OR AB(developing countries) OR TI(developing nation*) OR AB(developing nation*) OR TI(developing world) OR AB(developing world) OR TI(less-developed countr*) OR AB(less-developed countr*) OR TI(less developed countr*) OR AB(less developed countr*) OR TI(less-developed world) OR AB(less-developed world) OR TI(lesser developed world) OR AB(lesser developed world) OR TI(lesser-developed countr*) OR AB(lesser-developed countr*) OR TI(lesser developed countr*) OR AB(lesser developed countr*) OR TI(lesser-developed nation*) OR AB(lesser-developed nation*) OR TI(lesser developed nation*) OR AB(lesser developed nation*) OR TI(lesser-developed world) OR AB(lesser-developed world) OR TI(under-developed countr*) OR AB(under-developed countr*) OR TI(under developed countr*) OR AB(under developed countr*) OR TI(under-developed nation*) OR AB(under-developed nation*) OR TI(under developed nation*) OR AB(under developed nation*) OR TI(under-developed world) OR AB(under-developed world) OR TI(underdeveloped world) OR AB(underdeveloped world) OR TI(under developed world) OR AB(under developed world) OR TI(underdeveloped countr*) OR AB(underdeveloped countr*) OR TI(under-developed countr*) OR AB(under-developed countr*) OR TI(Under developed countr*) OR AB(Under developed countr*) OR TI(under developed nation*) OR AB(under developed nation*) OR TI(underdeveloped nation*) OR AB(underdeveloped nation*) OR TI(lower middle income country) OR AB(lower middle income country) OR TI(lower middle-income country) OR AB(lower middle-income country) OR TI(lower middle income nation) OR AB(lower middle income nation) OR TI(lower middle-income nation) OR AB(lower middle-income nation) OR TI(upper middle-income country) OR AB(upper middle-income country) OR TI(upper middle income country) OR AB(upper middle income country) OR TI(upper middle-income nation) OR AB(upper middle-income nation) OR TI(upper middle income nation) OR AB(upper middle income nation) OR TI(low-income country) OR AB(low-income country) OR TI(low income country) OR AB(low income country) OR TI(low-income nation) OR AB(low-income nation) OR TI(low income nation) OR AB(low income nation) OR TI(lower income country) OR AB(lower income country) OR TI(lower-income country) OR AB(lower-income country) OR TI(lower income nation) OR AB(lower income nation) OR TI(lower-income nation) OR AB(lower-income nation) OR TI(lmic) OR AB(lmic) OR TI(lmics) OR AB(lmics) OR TI(third world) OR AB(third world) OR TI(lami countr*) OR AB(lami countr*) OR TI(transitional countr*) OR AB(transitional countr*) OR TI(majority world) OR AB(majority world) OR TI(Global South) OR AB(Global South) OR TI(LMIC) OR AB(LMIC) OR TI(LMICs) OR AB(LMICs) OR TI(LIC) OR AB(LIC) OR TI(LICs) OR AB(LICs) OR TI(LMICs) OR AB(LMICs) OR TI(LMIC) OR AB(LMIC) OR TI(UMICs) OR AB(UMICs) OR TI(UMIC) OR AB(UMIC) OR TI(Afghanistan) OR AB(Afghanistan) OR TI(Burkina Faso) OR AB(Burkina Faso) OR TI(Burundi) OR AB(Burundi) OR TI(Central African Republic) OR AB(Central African Republic) OR TI(Chad) OR AB(Chad) OR TI(Congo, Dem.) OR AB(Congo, Dem.) OR TI(Eritrea) OR AB(Eritrea) OR TI(Ethiopia) OR AB(Ethiopia) OR TI(Gambia) OR AB(Gambia) OR TI(Guinea-Bissau) OR AB(Guinea-Bissau) OR TI(Korea, Dem. People's Rep) OR AB(Korea, Dem. People's Rep) OR TI(Liberia) OR AB(Liberia) OR TI(Madagascar) OR AB(Madagascar) OR TI(Malawi) OR AB(Malawi) OR TI(Mali) OR AB(Mali) OR TI(Mozambique) OR AB(Mozambique) OR TI(Niger) OR AB(Niger) OR TI(Rwanda) OR AB(Rwanda) OR TI(Sierra Leone) OR AB(Sierra Leone) OR TI(Somalia) OR AB(Somalia) OR TI(South Sudan) OR AB(South Sudan) OR TI(Sudan) OR AB(Sudan) OR TI(Syrian Arab Republic) OR AB(Syrian Arab Republic) OR TI(Togo) OR AB(Togo) OR TI(Uganda) OR AB(Uganda) OR TI(Yemen, Rep.) OR AB(Yemen, Rep.) OR TI(Angola) OR AB(Angola) OR TI(Algeria) OR AB(Algeria) OR TI(Bangladesh) OR AB(Bangladesh) OR TI(Benin) OR AB(Benin) OR TI(Bhutan) OR AB(Bhutan) OR TI(Bolivia) OR AB(Bolivia) OR TI(Cabo Verde) OR AB(Cabo Verde) OR TI(Cambodia) OR AB(Cambodia) OR TI(Cameroon) OR AB(Cameroon) OR TI(Comoros) OR AB(Comoros) OR TI(Congo, Rep.) OR AB(Congo, Rep.) OR TI(Côte d'Ivoire) OR AB(Côte d'Ivoire) OR TI(Djibouti) OR AB(Djibouti) OR TI(Egypt, Arab Rep.) OR AB(Egypt, Arab Rep.) OR TI(Eswatini) OR AB(Eswatini) OR TI(Ghana) OR AB(Ghana) OR TI(Guinea) OR AB(Guinea) OR TI(Haiti) OR AB(Haiti) OR TI(Honduras) OR AB(Honduras) OR TI(Jordan) OR AB(Jordan) OR TI(India) OR AB(India) OR TI(Iran, Islamic Rep) OR AB(Iran, Islamic Rep) OR TI(Kenya) OR AB(Kenya) OR TI(Kiribati) OR AB(Kiribati) OR TI(Kyrgyz Republic) OR AB(Kyrgyz Republic) OR TI(Lao PDR) OR AB(Lao PDR) OR TI(Lebanon) OR AB(Lebanon) OR TI(Lesotho) OR AB(Lesotho) OR TI(Mauritania) OR AB(Mauritania) OR TI(Micronesia, Fed. Sts.) OR AB(Micronesia, Fed. Sts.) OR TI(Mongolia) OR AB(Mongolia) OR TI(Morocco) OR AB(Morocco) OR TI(Myanmar) OR AB(Myanmar) OR TI(Nepal) OR AB(Nepal) OR TI(Nicaragua) OR AB(Nicaragua) OR TI(Nigeria) OR AB(Nigeria) OR TI(Pakistan) OR AB(Pakistan) OR TI(Papua New Guinea) OR AB(Papua New Guinea) OR TI(Philippines) OR AB(Philippines) OR TI(Samoa) OR AB(Samoa) OR TI(São Tomé) OR AB(São Tomé) OR TI(Principe) OR AB(Principe) OR TI(Senegal) OR AB(Senegal) OR TI(Solomon Islands) OR AB(Solomon Islands) OR TI(Sri Lanka) OR AB(Sri Lanka) OR TI(Tanzania) OR AB(Tanzania) OR TI(Tajikistan) OR AB(Tajikistan) OR TI(Timor-Leste) OR AB(Timor-Leste) OR TI(Tunisia) OR AB(Tunisia) OR TI(Ukraine) OR AB(Ukraine) OR TI(Uzbekistan) OR AB(Uzbekistan) OR TI(Vanuatu) OR AB(Vanuatu) OR TI(Vietnam) OR AB(Vietnam) OR TI(Zambia) OR AB(Zambia) OR TI(Zimbabwe) OR AB(Zimbabwe) OR TI(Albania) OR AB(Albania) OR TI(Argentina) OR AB(Argentina) OR TI(Armenia) OR AB(Armenia) OR TI(Azerbaijan) OR AB(Azerbaijan) OR TI(Belarus) OR AB(Belarus) OR TI(Belize) OR AB(Belize) OR TI(Bosnia) OR AB(Bosnia) OR TI(Herzegovina) OR AB(Herzegovina) OR TI(Botswana) OR AB(Botswana) OR TI(Brazil) OR AB(Brazil) OR TI(Bulgaria) OR AB(Bulgaria) OR TI(China) OR AB(China) OR TI(Colombia) OR AB(Colombia) OR TI(Costa Rica) OR AB(Costa Rica) OR TI(Cuba) OR AB(Cuba) OR TI(Dominica) OR AB(Dominica) OR TI(Dominican Republic) OR AB(Dominican Republic) OR TI(El Salvador) OR AB(El Salvador) OR TI(Equatorial Guinea) OR AB(Equatorial Guinea) OR TI(Ecuador) OR AB(Ecuador) OR TI(Fiji) OR AB(Fiji) OR TI(Gabon) OR AB(Gabon) OR TI(Georgia) OR AB(Georgia) OR TI(Grenada) OR AB(Grenada) OR TI(Guatemala) OR AB(Guatemala) OR TI(Indonesia) OR AB(Indonesia) OR TI(Iraq) OR AB(Iraq) OR TI(Jamaica) OR AB(Jamaica) OR TI(Kazakhstan) OR AB(Kazakhstan) OR TI(Kosovo) OR AB(Kosovo) OR TI(Libya) OR AB(Libya) OR TI(Malaysia) OR AB(Malaysia) OR TI(Maldives) OR AB(Maldives) OR TI(Marshall Islands) OR AB(Marshall Islands) OR TI(Mauritius) OR AB(Mauritius) OR TI(Mexico) OR AB(Mexico) OR TI(Moldova) OR AB(Moldova) OR TI(Montenegro) OR AB(Montenegro) OR TI(Namibia) OR AB(Namibia) OR TI(North Macedonia) OR AB(North Macedonia) OR TI(Palau) OR AB(Palau) OR TI(Paraguay) OR AB(Paraguay) OR TI(Peru) OR AB(Peru) OR TI(Russian Federation) OR AB(Russian Federation) OR TI(Serbia) OR AB(Serbia) OR TI(South Africa) OR AB(South Africa) OR TI(St. Lucia) OR AB(St. Lucia) OR TI(St. Vincent) OR AB(St. Vincent) OR TI(Suriname) OR AB(Suriname) OR TI(Thailand) OR AB(Thailand) OR TI(Tonga) OR AB(Tonga) OR TI(Türkiye) OR AB(Türkiye) OR TI(Turkmenistan) OR AB(Turkmenistan) OR TI(Tuvalu) OR AB(Tuvalu) OR TI(West Bank) OR AB(West Bank) OR TI(Gaza))  S2 - (TI(Community health care worker*) OR AB(Community health care worker*) OR TI(CHW*) OR AB(CHW*) OR TI(Community health worker*) OR AB(Community health worker*) OR TI(Front line health worker*) OR AB(Front line health worker*) OR TI(Outreach worker*) OR AB(Outreach worker*) OR TI(Lay health worker*) OR AB(Lay health worker*) OR TI(Lay counsellor*) OR AB(Lay counsellor*) OR TI(Health promoter*) OR AB(Health promoter*) OR TI(Village health worker*) OR AB(Village health worker*) OR TI(Volunteer health worker*) OR AB(Volunteer health worker*) OR TI(Community volunteer*) OR AB(Community volunteer*) OR TI(Village health volunteer*) OR AB(Village health volunteer*) OR TI(Lady health worker*) OR AB(Lady health worker*) OR TI(Barangay health worker*) OR AB(Barangay health worker*) OR TI(Outreach educator*) OR AB(Outreach educator*) OR TI(Shas*) OR AB(Shas*) OR TI(Shebika) OR AB(Shebika) OR TI(Shastho karmis) OR AB(Shastho karmis) OR TI(Village health helper*) OR AB(Village health helper*) OR TI(Accredited Social Health Activist, ASHA) OR AB(Accredited Social Health Activist, ASHA) OR TI(Family Health Worker*) OR AB(Family Health Worker*) OR TI(Peer Educator*) OR AB(Peer Educator*))  S1 - (TI(e-learning) OR AB(e-learning) OR TI(elearning) OR AB(elearning) OR TI(Electronic learning) OR AB(Electronic learning) OR TI(Online learning) OR AB(Online learning) OR TI(Distance learning) OR AB(Distance learning) OR TI(Massive Open Online Course) OR AB(Massive Open Online Course) OR TI(MOOC) OR AB(MOOC) OR TI(Mobile learning) OR AB(Mobile learning) OR TI(m-learning) OR AB(m-learning) OR TI(mlearning) OR AB(mlearning) OR TI(Webbased) OR AB(Webbased) OR TI(Web-based) OR AB(Web-based) OR TI(Offline learning) OR AB(Offline learning) OR TI(Offline distance learning) OR AB(Offline distance learning) OR TI(Technology enhanced learning) OR AB(Technology enhanced learning) OR TI(Digital innovations) OR AB(Digital innovations) OR TI(Hybrid learning) OR AB(Hybrid learning) OR TI(Flipped learning) OR AB(Flipped learning) OR TI(Blended education) OR AB(Blended education) OR TI(Multi-model learning) OR AB(Multi-model learning) OR TI(Hyflex learning) OR AB(Hyflex learning) OR TI(Asynchronous learning) OR AB(Asynchronous learning)) |
| EMBASE | #4 - #1 AND #2 AND #3  #3 - 'developing countr*':ab,ti OR 'developing countries':ab,ti OR 'developing nation*':ab,ti OR 'developing world':ab,ti OR 'less-developed countr*':ab,ti OR 'less developed countr*':ab,ti OR 'less-developed world':ab,ti OR 'lesser developed world':ab,ti OR 'lesser-developed countr*':ab,ti OR 'lesser developed countr*':ab,ti OR 'lesser-developed nation*':ab,ti OR 'lesser developed nation*':ab,ti OR 'lesser-developed world':ab,ti OR 'under-developed nation*':ab,ti OR 'under-developed world':ab,ti OR 'underdeveloped world':ab,ti OR 'under developed world':ab,ti OR 'underdeveloped countr*':ab,ti OR 'under-developed countr*':ab,ti OR 'under developed countr*':ab,ti OR 'under developed nation*':ab,ti OR 'underdeveloped nation*':ab,ti OR 'lower middle income country':ab,ti OR 'lower middle-income country':ab,ti OR 'upper middle-income country':ab,ti OR 'upper middle income country':ab,ti OR 'low-income country':ab,ti OR 'low income country':ab,ti OR 'lower income country':ab,ti OR 'lower-income country':ab,ti OR 'lower middle income countries':ab,ti OR 'lower middle-income countries':ab,ti OR 'lower middle income nation':ab,ti OR 'lower middle-income nation':ab,ti OR 'upper middle-income countries':ab,ti OR 'upper middle income countries':ab,ti OR 'upper middle-income nation':ab,ti OR 'upper middle income nation':ab,ti OR 'low income countries':ab,ti OR 'low-income nation':ab,ti OR 'low income nation':ab,ti OR 'lower income countries':ab,ti OR 'lower-income countries':ab,ti OR 'lower income nation':ab,ti OR 'lower-income nation':ab,ti OR 'third world':ab,ti OR 'lami countr*':ab,ti OR 'transitional countr*':ab,ti OR 'majority world':ab,ti OR 'global south':ab,ti OR 'lic':ab,ti OR 'lics':ab,ti OR 'lmics':ab,ti OR 'lmic':ab,ti OR 'umics':ab,ti OR 'umic':ab,ti OR 'afghanistan':ab,ti OR 'burkina faso':ab,ti OR 'burundi':ab,ti OR 'central african republic':ab,ti OR 'chad':ab,ti OR 'congo dem.':ab,ti OR 'eritrea':ab,ti OR 'ethiopia':ab,ti OR 'gambia':ab,ti OR 'guinea bissau':ab,ti OR 'korea dem peoples rep':ab,ti OR 'liberia':ab,ti OR 'madagascar':ab,ti OR 'malawi':ab,ti OR 'mali':ab,ti OR 'mozambique':ab,ti OR 'niger':ab,ti OR 'rwanda':ab,ti OR 'sierra leone':ab,ti OR 'somalia':ab,ti OR 'south sudan':ab,ti OR 'sudan':ab,ti OR 'syrian arab republic':ab,ti OR 'togo':ab,ti OR 'uganda':ab,ti OR 'yemen rep.':ab,ti OR 'angola':ab,ti OR 'algeria':ab,ti OR 'bangladesh':ab,ti OR 'benin':ab,ti OR 'bhutan':ab,ti OR 'bolivia':ab,ti OR 'cabo verde':ab,ti OR 'cambodia':ab,ti OR 'cameroon':ab,ti OR 'comoros':ab,ti OR 'congo rep.':ab,ti OR 'côte divoire':ab,ti OR 'djibouti':ab,ti OR 'egypt arab rep.':ab,ti OR 'eswatini':ab,ti OR 'ghana':ab,ti OR 'guinea':ab,ti OR 'haiti':ab,ti OR 'honduras':ab,ti OR 'jordan':ab,ti OR 'india':ab,ti OR 'iran islamic rep':ab,ti OR 'kenya':ab,ti OR 'kiribati':ab,ti OR 'kyrgyz republic':ab,ti OR 'lao pdr':ab,ti OR 'lebanon':ab,ti OR 'lesotho':ab,ti OR 'mauritania':ab,ti OR 'micronesia, fed. sts.':ab,ti OR 'mongolia':ab,ti OR 'morocco':ab,ti OR 'myanmar':ab,ti OR 'nepal':ab,ti OR 'nicaragua':ab,ti OR 'nigeria':ab,ti OR 'pakistan':ab,ti OR 'papua new guinea':ab,ti OR 'philippines':ab,ti OR 'samoa':ab,ti OR 'são tomé':ab,ti OR 'principe':ab,ti OR 'senegal':ab,ti OR 'solomon islands':ab,ti OR 'sri lanka':ab,ti OR 'tanzania':ab,ti OR 'tajikistan':ab,ti OR 'timor leste':ab,ti OR 'tunisia':ab,ti OR 'ukraine':ab,ti OR 'uzbekistan':ab,ti OR 'vanuatu':ab,ti OR 'vietnam':ab,ti OR 'zambia':ab,ti OR 'zimbabwe':ab,ti OR 'albania':ab,ti OR 'argentina':ab,ti OR 'armenia':ab,ti OR 'azerbaijan':ab,ti OR 'belarus':ab,ti OR 'belize':ab,ti OR 'bosnia':ab,ti OR 'herzegovina':ab,ti OR 'botswana':ab,ti OR 'brazil':ab,ti OR 'bulgaria':ab,ti OR 'china':ab,ti OR 'colombia':ab,ti OR 'costa rica':ab,ti OR 'cuba':ab,ti OR 'dominica':ab,ti OR 'dominican republic':ab,ti OR 'el salvador':ab,ti OR 'equatorial guinea':ab,ti OR 'ecuador':ab,ti OR 'fiji':ab,ti OR 'gabon':ab,ti OR 'georgia':ab,ti OR 'grenada':ab,ti OR 'guatemala':ab,ti OR 'indonesia':ab,ti OR 'iraq':ab,ti OR 'jamaica':ab,ti OR 'kazakhstan':ab,ti OR 'kosovo':ab,ti OR 'libya':ab,ti OR 'malaysia':ab,ti OR 'maldives':ab,ti OR 'marshall islands':ab,ti OR 'mauritius':ab,ti OR 'mexico':ab,ti OR 'moldova':ab,ti OR 'montenegro':ab,ti OR 'namibia':ab,ti OR 'north macedonia':ab,ti OR 'palau':ab,ti OR 'paraguay':ab,ti OR 'peru':ab,ti OR 'russian federation':ab,ti OR 'serbia':ab,ti OR 'south africa':ab,ti OR 'st lucia':ab,ti OR 'st vincent':ab,ti OR 'suriname':ab,ti OR 'thailand':ab,ti OR 'tonga':ab,ti OR 'türkiye':ab,ti OR 'turkmenistan':ab,ti OR 'tuvalu':ab,ti OR 'west bank':ab,ti OR 'gaza':ab,ti  #2 - 'community health care worker*':ab,ti OR 'chw*':ab,ti OR 'community health worker*':ab,ti OR 'front line health worker*':ab,ti OR 'outreach worker*':ab,ti OR 'lay health worker*':ab,ti OR 'lay counsellor*':ab,ti OR 'health promoter*':ab,ti OR 'village health worker*':ab,ti OR 'volunteer health worker*':ab,ti OR 'community volunteer*':ab,ti OR 'village health volunteer*':ab,ti OR 'lady health worker*':ab,ti OR 'barangay health worker*':ab,ti OR 'outreach educator*':ab,ti OR 'shas*':ab,ti OR 'shebika':ab,ti OR 'shastho karmis':ab,ti OR 'village health helper*':ab,ti OR 'accredited social health activist, asha':ab,ti OR 'family health worker*':ab,ti OR 'peer educator*':ab,ti  #1 - 'virtual learning':ab,ti OR 'e-learning':ab,ti OR 'elearning':ab,ti OR 'electronic learning':ab,ti OR 'online learning':ab,ti OR 'distance learning':ab,ti OR 'massive open online course':ab,ti OR 'mooc':ab,ti OR 'mobile learning':ab,ti OR 'm-learning':ab,ti OR 'mlearning':ab,ti OR 'webbased':ab,ti OR 'web-based':ab,ti OR 'offline learning':ab,ti OR 'offline distance learning':ab,ti OR 'technology enhanced learning':ab,ti OR 'digital innovations':ab,ti OR 'hybrid learning':ab,ti OR 'flipped learning':ab,ti OR 'blended education':ab,ti OR 'multi-model learning':ab,ti OR 'hyflex learning':ab,ti OR 'asynchronous learning':ab,ti |
| ERIC | S4 - S1 AND S2 AND S3  S3 - ("developing countr*" OR "developing countries" OR "developing nation*" OR "developing world" OR "less-developed countr*" OR "less developed countr*" OR "less-developed world" OR "lesser developed world" OR "lesser-developed countr*" OR "lesser developed countr*" OR "lesser-developed nation*" OR "lesser developed nation*" OR "lesser-developed world" OR "under-developed countr*" OR "under developed countr*" OR "under-developed nation*" OR "under developed nation*" OR "under-developed world" OR "underdeveloped world" OR "under developed world" OR "underdeveloped countr*" OR "under-developed countr*" OR "Under developed countr*" OR "under developed nation*" OR "underdeveloped nation*" OR "lower middle income country" OR "lower middle-income country" OR "lower middle income nation" OR "lower middle income nation" OR "upper middle-income country" OR "upper middle income country" OR "upper middle-income nation" OR "upper middle income nation" OR "low-income country" OR "low income country" OR "low-income nation" OR "low income nation" OR "lower income country" OR "lower-income country" OR "lower income nation" OR "lower-income nation" OR "lower middle income countries" OR "lower middle-income countries" OR "lower middle income nation" OR "lower middle-income nation" OR "upper middle-income countries" OR "upper middle income countries" OR "upper middle-income nation" OR "upper middle income nation" OR "low income countries" OR "low income countries" OR "low-income nation" OR "low income nation" OR "lower income countries" OR "lower-income countries" OR "lower income nation" OR "lower-income nation" OR "lmic" OR "lmics" OR "third world" OR "lami countr*" OR "transitional countr*" OR "majority world" OR "Global South" OR "LMIC" OR "LMICs" OR "LIC" OR "LICs" OR "LMICs" OR "LMIC" OR "UMICs" OR "UMIC" OR "Afghanistan" OR "Burkina Faso" OR "Burundi" OR "Central African Republic" OR "Chad" OR "Congo, Dem." OR "Eritrea" OR "Ethiopia" OR "Gambia" OR "Guinea-Bissau" OR "Korea, Dem. People's Rep" OR "Liberia" OR "Madagascar" OR "Malawi" OR "Mali" OR "Mozambique" OR "Niger" OR "Rwanda" OR "Sierra Leone" OR "Somalia" OR "South Sudan" OR "Sudan" OR "Syrian Arab Republic" OR "Togo" OR "Uganda" OR "Yemen, Rep." OR "Angola" OR "Algeria" OR "Bangladesh" OR "Benin" OR "Bhutan" OR "Bolivia" OR "Cabo Verde" OR "Cambodia" OR "Cameroon" OR "Comoros" OR "Congo, Rep." OR "Côte d'Ivoire" OR "Djibouti" OR "Egypt, Arab Rep." OR "Eswatini" OR "Ghana" OR "Guinea" OR "Haiti" OR "Honduras" OR "Jordan" OR "India" OR "Iran, Islamic Rep" OR "Kenya" OR "Kiribati" OR "Kyrgyz Republic" OR "Lao PDR" OR "Lebanon" OR "Lesotho" OR "Mauritania" OR "Micronesia, Fed. Sts." OR "Mongolia" OR "Morocco" OR "Myanmar" OR "Nepal" OR "Nicaragua" OR "Nigeria" OR "Pakistan" OR "Papua New Guinea" OR "Philippines" OR "Samoa" OR "São Tomé" OR "Principe" OR "Senegal" OR "Solomon Islands" OR "Sri Lanka" OR "Tanzania" OR "Tajikistan" OR "Timor-Leste" OR "Tunisia" OR "Ukraine" OR "Uzbekistan" OR "Vanuatu" OR "Vietnam" OR "Zambia" OR "Zimbabwe" OR "Albania" OR "Argentina" OR "Armenia" OR "Azerbaijan" OR "Belarus" OR "Belize" OR "Bosnia" OR "Herzegovina" OR "Botswana" OR "Brazil" OR "Bulgaria" OR "China" OR "Colombia" OR "Costa Rica" OR "Cuba" OR "Dominica" OR "Dominican Republic" OR "El Salvador" OR "Equatorial Guinea" OR "Ecuador" OR "Fiji" OR "Gabon" OR "Georgia" OR "Grenada" OR "Guatemala" OR "Indonesia" OR "Iraq" OR "Jamaica" OR "Kazakhstan" OR "Kosovo" OR "Libya" OR "Malaysia" OR "Maldives" OR "Marshall Islands" OR "Mauritius" OR "Mexico" OR "Moldova" OR "Montenegro" OR "Namibia" OR "North Macedonia" OR "Palau" OR "Paraguay" OR "Peru" OR "Russian Federation" OR "Serbia" OR "South Africa" OR "St. Lucia" OR "St. Vincent" OR "Suriname" OR "Thailand" OR "Tonga" OR "Türkiye" OR "Turkmenistan" OR "Tuvalu" OR "West Bank" OR "Gaza")  S2 - ("Community health care worker*" OR "CHW*" OR "Community health worker*" OR "Front line health worker*" OR "Outreach worker*" OR "Lay health worker*" OR "Lay counsellor*" OR "Health promoter*" OR "Village health worker*" OR "Volunteer health worker*" OR "Community volunteer*" OR "Village health volunteer*" OR "Lady health worker*" OR "Barangay health worker*" OR "Outreach educator*" OR "Shas*" OR "Shebika" OR "Shastho karmis" OR "Village health helper*" OR "Accredited Social Health Activist, ASHA" OR "Family Health Worker*" OR "Peer Educator*")  S1 - ("Virtual learning" OR "e-learning" OR "elearning" OR "Electronic learning" OR "Online learning" OR "Distance learning" OR "Massive Open Online Course" OR "MOOC" OR "Mobile learning" OR "m-learning" OR "mlearning" OR "Webbased" OR "Web-based" OR "Offline learning" OR "Offline distance learning" OR "Technology enhanced learning" OR "Digital innovations" OR "Hybrid learning" OR "Flipped learning" OR "Blended education" OR "Multi-model learning" OR "Hyflex learning" OR "Asynchronous learning") |
| Global Health | (("developing countr*" OR "developing nation*" OR "developing world" OR "less* developed countr*" OR "underdeveloped countr*" OR "low-income countr*" OR "lower-middle-income countr*" OR "upper-middle-income countr*" OR LMIC OR LMICs OR LIC OR LICs OR UMIC OR UMICs OR "third world" OR "Global South" OR "resource-limited setting*" OR "emerging econom*" OR Afghanistan OR "Burkina Faso" OR Burundi OR "Central African Republic" OR Chad OR Eritrea OR Ethiopia OR Gambia OR Malawi OR Mozambique OR Niger OR Rwanda OR Somalia OR Sudan OR Togo OR Uganda OR "Yemen, Rep." OR Angola OR Algeria OR Bangladesh OR Bhutan OR Bolivia OR Cambodia OR Cameroon OR Djibouti OR Egypt OR Ghana OR Guinea OR Haiti OR India OR Indonesia OR Kenya OR Nigeria OR Pakistan OR Philippines OR Senegal OR Tanzania OR Ukraine OR Zambia OR Zimbabwe OR Albania OR Argentina OR Armenia OR Brazil OR China OR Colombia OR Mexico OR "South Africa" OR Thailand OR Türkiye)) AND (("Community health care worker" OR CHW OR "Community health worker" OR "Front line health worker" OR "Outreach worker" OR "Lay health worker" OR "Lay counsellor" OR "Health promoter" OR "Village health worker" OR "Volunteer health worker" OR "Community volunteer" OR "Village health volunteer" OR "Lady health worker" OR "Barangay health worker" OR "Outreach educator" OR Shas OR Shebika OR "Shastho karmis" OR "Village health helper" OR "Accredited Social Health Activist" OR ASHA OR "Family Health Worker" OR "Peer Educator")) AND (("Virtual learning" OR "e-learning" OR elearning OR "Electronic learning" OR "Online learning" OR "Distance learning" OR "Massive Open Online Course" OR MOOC OR "Mobile learning" OR "m-learning" OR mlearning OR Webbased OR "Web-based" OR "Offline learning" OR "Offline distance learning" OR "Technology enhanced learning" OR "Digital innovations" OR "Hybrid learning" OR "Flipped learning" OR "Blended education" OR "Multi-model learning" OR "Hyflex learning" OR "Asynchronous learning")) |
| COCHRANE | (("Virtual learning" OR "e-learning" OR "elearning" OR "Electronic learning" OR "Online learning" OR "Distance learning" OR "Massive Open Online Course" OR "MOOC" OR "Mobile learning" OR "m-learning" OR "mlearning" OR "Webbased" OR "Web-based" OR "Offline learning" OR "Offline distance learning" OR "Technology enhanced learning" OR "Digital innovations" OR "Hybrid learning" OR "Flipped learning" OR "Blended education" OR "Multi-modal learning" OR "Hyflex learning" OR "Asynchronous learning" OR "Digital Training" OR "Digital learning" OR "Virtual training" OR "Online education" OR "Remote training")) AND (("Community health care worker" OR "CHW" OR "Community health worker" OR "Frontline health worker" OR "Outreach worker" OR "Lay health worker" OR "Lay counsellor" OR "Health promoter" OR "Village health worker" OR "Volunteer health worker" OR "Community volunteer" OR "Village health volunteer" OR "Lady health worker" OR "Barangay health worker" OR "Outreach educator" OR "Shas" OR "Shebika" OR "Shastho karmis" OR "Village health helper" OR "Accredited Social Health Activist" OR "ASHA" OR "Family Health Worker" OR "Peer Educator" OR "Health extension worker" OR "Community outreach worker" OR "Village volunteer")) in Title Abstract Keyword - in Cochrane Reviews, Cochrane Protocols, Trials, Clinical Answers, Editorials, Special Collections |
| GOOGLE SCHOLAR | allintitle:("blended learning" OR "digital training" OR "e learning" OR "online courses" OR webinars) AND ("lay health care worker" OR "health provider" OR "health worker" OR "health volunteers" OR "community health worker" OR "health extension workers" OR "health care" OR "primary health care worker" OR "primary care provider" OR "public health worker" OR "lay health worker" OR "peer support worker" OR "lay counselor" OR "community health aide" OR "health promoter" OR "frontline health worker" OR "village health worker" OR "lady health worker" OR "barefoot doctor" OR "health educator" OR "health facilitator" OR "health advisor" OR "health navigator" OR "health liaison" OR "outreach worker" OR "health assistant" OR "health guide") |
| SSRN | ("Blended learning" OR "e-learning") AND ("Health") |

**Table S2. Individual database search results**

| Database | Number of hits | Page number for search  strategy details |
| --- | --- | --- |
| PubMed | 56 | 1-4 |
| CINHAL | 19 | 4-7 |
| SCOPUS | 108 | 7-9 |
| PsychINFO | 112 | 9-12 |
| EMBASE | 83 | 12-14 |
| ERIC | 27 | 14-16 |
| Global Health | 23 | 16 |
| COCHRANE | 34 | 16-17 |
| GOOGLE SCHOLAR | 418 | 17 |
| SSRN | 12 | 17 |

Legend: The results from individual database searches, including the number of hits and the supplementary material page numbers where the search strategies can be found.
